# Small Changes, But Huge Impact? The Right Anterior Insula's Loss of Connection Strength during the Transition of Old to Very Old Age

**DOI:** 10.3389/fnagi.2016.00086

**Published:** 2016-05-10

**Authors:** Angela M. Muller, Susan Mérillat, Lutz Jäncke

**Affiliations:** ^1^University Research Priority Program, Dynamics of Healthy Aging, University of ZurichZurich, Switzerland; ^2^International Normal Aging and Plasticity Imaging Center, University of ZurichZurich, Switzerland; ^3^Division Neuropsychology, Institute of Psychology, University of ZurichZurich, Switzerland; ^4^Center for Integrative Human Physiology, University of ZurichZurich, Switzerland

**Keywords:** healthy aging, resting-state, executive-control network, salience network, default-mode, deactivation, network configurations

## Abstract

A major contribution to our understanding of the aging brain comes either from studies comparing young with older adults or from studies investigating pathological aging and using the healthy aging older adults as control group. In consequence, we know relatively well, what distinguishes young from old brains or pathological aging from healthy but that does not mean that we really understand the structural and functional transformations characterizing the healthy aging brain. By analyzing task-free fMRI data from a large cross-sectional sample of 186 older adults (mean age = 70.4, 97 female), we aimed to elucidate age-related changes in the intrinsically active functional architecture of the brain in our study group covering an age range from 65 to 85 years. First, we conducted an intrinsic connectivity contrast analysis (ICC) in order to detect the brain regions whose degree of connectedness was significantly correlated with increasing age. Secondly, using connectivity analyses we investigated how the clusters highlighted by the ICC analysis functionally related to the other major resting-state networks. The most important finding was the right anterior insula's loss of connectedness in the older participants of the study group because of the region's causal role in the switching from the task-negative to the task-positive state of the brain. Further, we found a higher functional dedifferentiation of two of the brain's major intrinsic connectivity networks, the DMN, and the cingulo-opercular network, caused by a reduction of functional connection strength, especially in the frontal regions. At last, we showed that all these age-related changes have the potential to impair older adult's performance of working memory tasks.

## Introduction

Growing old is not only associated with the aging brain's structural degeneration like progressive gray matter atrophy or loss of white matter integrity as evidenced by structural neuroimaging methods (Raz et al., 1997; Good et al., 2001; Sowell et al., 2003; Salat et al., 2004; Fjell et al., 2009; Jäncke et al., 2015) but also with cognitive and behavioral changes. Functional neuroimaging methods like PET or fMRI, on the other hand, were able to provide a slightly different point of view: Despite manifest structural decline, there is evidence for functional reorganization of the aging brain. It has repeatedly been observed that activation patterns of older brains are spatially more extensive in comparison to the activation patterns of younger participants even though young and old still perform at the same level. In addition, older adults seem to recruit additional brain regions. However, while task-induced fMRI studies investigating the aging brain generally observe roughly the same age-typical changes in the BOLD-signal patterns, the interpretations of these age-related patterns can be quite different, ranging from functional dedifferentiation (Persson et al., 2007; Meinzer et al., 2012; Berlingeri et al., 2013) to successful compensation (Cabeza, 2002; Davis et al., 2008).

About a decade ago, there was a paradigm shift in how these activation patterns are interpreted; away from a rather localization-oriented understanding that an area showing a significant increase in activity during the task has to be the brain region subserving just that specific function to a more network-oriented perspective. Thus, not a single brain area is thought to be responsible for a specific function but rather a coordinated interaction of a group of brain regions forming a network or even a cooperation of different functional networks, all interacting with each other in a fine-tuned concerted manner. Studies using different forms of connectivity analyses, analyzing task-induced as well as task-free fMRI data, were able to show that the aging brain can generally be characterized by reduced functional connection strengths in comparison to the brain of younger adults. This affects the entire functional architecture of the brain. Not only the within-network connections decrease but also the between-network connections of the brain are affected and the different networks eventually seem to merge into each other and become more and more functionally dedifferentiated with age (Meunier et al., 2009; Betzel et al., 2014; Geerligs et al., 2014).

Up to now, the knowledge about age-related alterations of the brain mainly comes from two branches of research. First, there are a quite impressive number of cross-sectional studies comparing young with older adults. However, a limitation of the cross-sectional study design is that one is looking at both age groups as two quite homogeneous entities, i.e., young vs. old, and thereby neglecting the inner-group variance. Especially in groups of older subjects, inter-individual differences can be large and people develop along different trajectories ranging from pathologically aging to healthy aging. Therefore, although we now have gained some knowledge about the differences between young and older brains, we still do not very well understand how age-related structural, functional and cognitive changes are related to each other and producing such a variety of aging trajectories even in the healthy aging older adults. The second line of research focuses on understanding the mechanism that underlies pathological aging. Here, the brain and behavioral data of healthy older adults are used in order to highlight the differences between disease and health, but the processes of healthy aging are not in the main interest. Consequently, one might consider understanding the mechanisms characterizing the healthy aging brain, i.e., understanding the changes of the aging brain from old to very old age and the implications for behavior and cognition, as a field of research still deserving more interest and effort.

The aim of the current study was to elucidate the age-related differences of the brain's functional architecture in a study group covering the age range from healthy old age (>65 years) to very old healthy age (>80 years) and to relate these functional differences to actual cognitive performance. We used task-free fMRI data because we wanted to eliminate all possible confounding factors, including the generally higher testing anxiety in older participants in a MRI scanner, the difficulty for older participants to gain familiarity with the artificial testing designs that are often unavoidable in fMRI experiments, the increased variation in the problem-solving strategies of older participants, the testing fatigue that usually occurs more quickly in older participants, and many other factors, all of which can heavily influence the findings of task-induced fMRI paradigms in older adults.

In order to be as unbiased as possible for the question at hand, we also decided in favor of a purely data driven analysis. Therefore, in a first step, we performed an Intrinsic Connectivity Contrast (ICC) analysis (Martuzzi et al., 2011). The ICC analysis is a whole-brain analysis method on the voxel-level that is able to identify the brain regions whose degree of connectedness with other brain regions is associated with increasing age without the need to determine a priori regions of interest. In a second step, we partitioned the brain into functionally defined clusters and then computed a connectivity analysis with the ICC clusters as source regions in order to specify their most relevant target regions. In a third step we correlated the resulting connectivity profile of each ICC cluster with age and with the participants' performance in working memory for the purpose of elucidating how the age-related alterations in the intrinsically active baseline configuration are associated with performance in a cognitive function, which is very well established to be affected by the aging process (Draganski et al., 2014).

## Methods

### Participants

All MRI and behavioral data used in the study presented here were acquired in the context of the first data collection time-point of the Longitudinal Healthy Aging Brain (LHAB) project. The LHAB is a longitudinal database project that started in 2011 at the International Normal Aging and Plasticity Imaging Center (INAPIC) of the University of Zurich is currently run at the University Research Priority Program (URPP) “Dynamics of Healthy Aging” (Zöllig et al., 2011). By combining extensive cognitive testing with thorough MRI exams, the LHAB project aspires to investigate and understand the cognitive and neural mechanisms that promote healthy aging of the brain.

At every point of measurement, the participants of the LHAB-project have to undergo an MRI exam of 1 h and to take an intensive cognitive assessment. The LHAB test-battery consists of different neuropsychological tests in reasoning (3), verbal intelligence (3), verbal fluency (2), executive functions (3), processing speed (3), attention (3), working memory (3), memory (3), spatial orientation (3), spatial reasoning (4), and motoric and fine motoric coordination (3). Additionally, the participants have to complete questionnaires about physical and mental health, medication, sleep quality and day drowsiness, affect, mood, and their social networks.

To qualify for participation in the LHAB project, interested persons had to fulfill the following criteria: Suitability for MRI assessments, 65 years or older, right handed, native Swiss-German or native German speakers and healthy, i.e., no history or current diagnosis of psychiatric or neurologic diseases, e.g., Parkinson's disease, Alzheimer's dementia, multiple sclerosis, migraine, further a MMSE score of >26 and no chronic or acute medical conditions such as diabetes, tinnitus, and diseases of the hematopoietic system. All participants gave written informed consent to the participation of the longitudinal project under the approval of the local ethics committee, the Kantonale Ethikkommission Zurich, and in accordance with the declaration of Helsinki.

**Figure 1:**
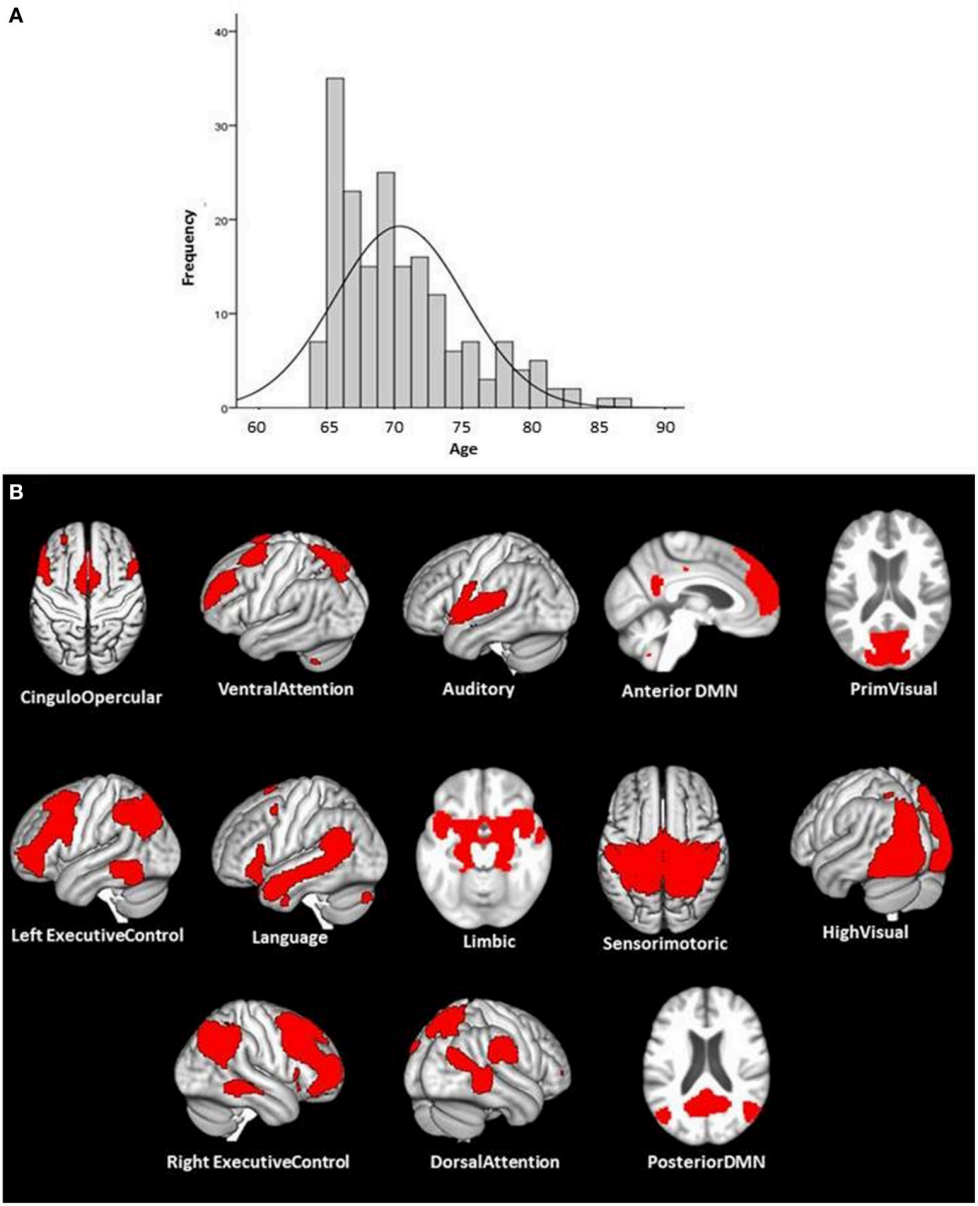
**The age-distribution of the study sample and the illustration of the 13 ICA components that could be identified as functional meaningful and well-established ICNs**. The Panel **(A)** illustrates the age distribution of the study sample (*n* = 186; mean age = 70.4, 97 female and 89 male). The Panel **(B)** shows the illustration of the 13 used ICA components used to partition the brain into functional networks.

The baseline measurement of the LHAB project started in 2011 and was finalized in 2013, the data of 230 participants were acquired during this period. However, we had to exclude the data of 9 participants because of incompleteness of the MRI data, 32 due to motion artifacts over 2 mm, one because of pathological high white matter lesion load, and one due to a diagnosis of type II diabetes shortly after the first time of assessment. Finally, the data of two other participants were not analyzed because they fulfilled the defined outlier criteria for the cognitive tests by scoring significantly lower (≥3 SD) than the rest of the study sample in 10% of the overall 115 cognitive tests and subtests. Consequently, we were able to use the data of 186 subjects (mean age = 70.4; SD = 4.8; 89 men and 97 women, mean years of education = 14.6; SD = 3.46) for the study presented here. A two sample *t*-test confirmed that there was no significant age difference between the remaining 186 participants and the excluded 44 subjects. However, as the LHAB database was especially designed to fulfill the specific requirements of a longitudinal analysis approach, the age distribution of the 186 subjects might be not so ideal for a cross-sectional approach that was used for the study presented here. The age distributions showed a distinct predominance of participants (105 of the 186) covering a relatively small age range from 65 to 70 years. As a result, subjects aged 75 or older (= 32 participants) might be relatively underrepresented in the study sample (Figure 1A).

### MRI-data acquisition

All MRI-data were acquired on the same Philips 3T Ingenia Medical Scanner with a Philips 15 channel head-coil at the University Hospital Zurich.

From the LHAB MRI protocol, the following images were used: (1) A T1 weighted TFE- SENSE sequence with TR = 8.2 ms, TE = 3.7 ms, Flip Angle = 90 (FOV = 240 × 160 × 240 mm^3^, matrix size = 256 × 256, isotropic voxel size = 0.94 × 0.94 × 1 mm) 160 slices per volume. (2) A task-free blood oxygenation level-dependent signals (BOLD) single shot whole brain EPI with a TR = 2000 ms, TE = 21 ms, Flip Angle = 76; 43 transverse slices (FOV = 220 × 150 × 220 mm^3^, matrix size = 64 × 64, ascending acquisition without gap, anisotropic voxel size = 1.72/1.72/3.50 mm, aligned to the AC-PC line), acquisition time 7.39 min, 225 volumes.

For this part of the data acquisition, the participants were instructed to lay as motionless as possible, stay awake and think of nothing in particular while fixating a cross that was displayed on a screen outside the scanner and was visible for the participant by means of a mirror system attached to the receiver coil. Immediately after the task-free scan, each participant had to confirm that he/she had not fallen asleep during that part of the MRI exams.

### Pre-processing of the fMRI data

All pre-processing steps of the MRI data listed below were performed using the SPM12 software (http://www.fil.ion.ucl.ac.uk/spm) implemented in Matlab (MATLAB R2014b, The Math Works Inc.).

First, the first six volumes of the task-free fMRI data were discarded allowing for T1 saturation effects, leaving 219 volumes for the analysis and then realigned in a two-pass procedure to correct for potential head movements during the task-free scan. Second, the fMRI time series were slice time corrected for the ascending acquisition. Third, the T1 weighted anatomical image was coregistered to the mean functional image generated during the alignment step. Fourth, the T1 weighted anatomical images were segmented into the three tissue classes, gray matter, white matter, and cerebrospinal fluid in native space. Fifth, a study-population specific GM template was generated using the DARTEL routine (Ashburner, 2007) implemented in SPM12 that allows for a high dimensional and nonlinear registration of the anatomical and functional images and their subsequent normalization to the MNI-template. The functional and the anatomical data subsequently used for the functional analyses were resampled to a 2 mm isotropic voxel-size during this step. While the functional images for the ICC analysis were only minimally smoothed using an isotropic Gaussian kernel (FWHM 1 mm) in order to minimize possible spurious correlations, the functional images used in ICA were smoothed using an isotropic Gaussian kernel (FWHM 6 mm).

### First analysis step: intrinsic connectivity contrast (ICC) analyses on the pre-processed task free fMRI data

All following intrinsic connectivity (IC) analyses were performed using the Conn Toolbox (version 15a) (http://www.nitrc.org/projects/conn) for SPM (Whitfield-Gabrieli and Nieto-Castanon, 2012).

Band-pass filtering and denoising of the data: The BOLD signal time-series for each participant was extracted and band-pass-filtered (0.008—0.09 Hz), as well as despiked and detrended. Non-neural low frequent (< 0.1 Hz) signals such as heart rate or respiration are able to modulate the intrinsic low frequent fluctuations of the BOLD-signal, which are the substructure investigated in task-free fMRI analysis. Therefore, it is important to correct for these physiological confounding signals. The Conn Toolbox accomplishes this by means of the CompCor method (Behzadi et al., 2007). As the final step during the denoising procedure implemented in the Conn Toolbox the motion parameters as well as the confounding signals calculated by the CompCor method are regressed out.

First level analyses: The Intrinsic Connectivity Contrast Power (ICCp) index (Martuzzi et al., 2011) is a whole brain voxel based measure of connectivity related to the graph theory analysis (GTA) measure “*degree*” (Rubinov and Sporns, 2010). Compared to the standard GTA methods an ICC analysis has two advantages: (1) An ICCp analysis is data driven and free of any need to define a seed based on a priori assumptions about regions of interest. (2) The ICCp analysis does not depend on arbitrarily thresholding the network because the index works by weighting the connections of a voxel with the other voxels by the strength of these connections, respectively, by the square number of its correlation strength, and therefore producing voxel based connectivity maps reflecting the existence as well as the strength of the connections of every individual voxel (Martuzzi et al., 2011).

On the second level or group level, the Conn Toolbox computes a GLM over all participants. To compute the connectivity maps illustrating the voxel level ICCp index and its correlation with age on group level, a high threshold for the peak voxels of *p* < 0.01 uncorrected, and an extension threshold for the clusters of *p* < 0.05 FWE corrected was used. At this point, we want to emphasize that we are not interpreting the results of the ICCp analyses in terms of statistical significance, but we are interpreting the *p*-values as a measure of an effect, i.e., as a sort of threshold for defining sensible seized ROIs, which can be used in the following connectivity analyses. By doing this, we refer to Krauth (1988), who recommended using “the *p*-value as the lowest significance level at which someone would still have obtained a significant result for a given data set, a given significance test, and a given test problem. This has the advantage that other researchers can decide for themselves whether the results are significant at the significance level they may find acceptable.”

### Second analysis step: seed correlation analyses to identify the functional network affiliation of the ICCp clusters

The ICCp maps of the first analysis step identified the clusters whose ICCp index was significantly correlated with age. The next step was to establish the intrinsic connectivity network (ICN) affiliation of each of these regions highlighted by the ICCp analysis by means of a subsequent seed correlations analysis (SCA).

To this end, correlation maps on voxel-level were computed by correlating the BOLD-signal time-course, averaged over all voxels constituting an ICCp cluster, with the BOLD-signal time-course of every other single voxel over the duration of the task-free scan for each participant.

For the group-level analysis the toolbox Conn implements contrasts for analyses at the voxel level as repeated-measures analyses by using ReML estimation of covariance components that are evaluated through F-statistical parameter maps. A height threshold for the peak voxel: *p* < 0.001 FDR corrected and an extension threshold for the clusters: *p* < 0.001 FWE corrected were used for the computing of the intrinsic connectivity map for the ICC cluster.

### Third analysis step: independent component analysis (ICA) to partition the brain into functionally relevant networks and ROIs

Results of an ICC analysis can only highlight regions that demonstrate an association of connectedness with a specific feature like different states of consciousness, cognitive performance, or, as in this case, with increasing age. To identify which of the numerous connections a given ICCp cluster maintains with other brain regions have exactly triggered the ICCp findings additional analyses are necessary. Therefore, to get an understanding of how the ICCp clusters relate beyond their own network to other ICN's of the brain, we performed an ICA in order to partition the brain into functionally meaningful networks and ROIs that are associated with these networks.

For this step, the functional images already smoothed with an isotropic Gaussian kernel of 6 mm (FWHM) during the preprocessing were additionally band-pass-filtered (0.008–0.09 Hz) and linearly detrended using the DPARS-F Toolbox (Chao-Gan and Yu-Feng, 2010).

For the ICA we used the MATLAB based Group ICA of fMRI Toolbox (GIFT) v3.0a (http://mialab.mrn.org/software/gift/). ICA is a statistical method of blind signal source separation. Assuming a generative model and a linear mixture of independent sources, it works with higher order statistics in order to maximize the spatial or temporal independence of the data and to identify the independent components hidden in the signal (Calhoun et al., 2001). In order to get components preferably representing whole networks instead of single ROIs or sub-networks we used a low dimensional approach and extracted 30 components using the Infomax algorithm. Of the extracted 30 components, 13 components could be identified as neurophysiologically relevant ICNs by visual inspection and by comparing them with the results of previous studies that likewise used a low-dimensional ICA approach to parcellate the brain into ICNs (Damoiseaux et al., 2006; Shirer et al., 2012). In the next step, we thresholded the ICA components at a *z*-value = 5 for the purpose of getting a sensible number of ROIs as well as reasonable sized ROIs and separated the components into their constituent single clusters using mricron (http://www.mccauslandcenter.sc.edu/mricro/index.html).

At last, we additionally computed the association of each of the 13 networks with the age of the participants in order to test for potential age-effects on the within network architecture of these networks.

### Fourth analysis step: connectivity analyses to investigate the relationship of the ICCP clusters among the other functional intrinsic connectivity networks (ICN)

To elucidate how the ICCp clusters relate to the other 13 ICNs identified by the ICA, the former were defined as source ROIs while the 93 ICA-derived clusters were defined as target ROIs

#### Age effects in the functional connection profile of the ICC clusters showing age-related alterations in connectedness

In a first step we computed the connection profile of each ICCp cluster for the purpose of understanding how the ICCp clusters generally relate to the 13 networks and 93 network ROIs identified by the previous ICA. In a next step, we correlated the connection strengths of the connection profiles computed for each ICCp cluster in the first step with the age of the participants in order to bring out the connections specifically affected by age-related differences in connectedness.

#### Additional steps to ensure the independency between search and test

We are well aware of the fact that we computed circular analyses by correlating the connection profiles of the ICCp clusters with the participants' age a second time. The reasons of this course of action were as follows: (1) As mentioned before in the Methods Section, the age distribution of the study sample used in this paper was characterized by a majority of participants representing a relatively small age range from 65 to 70 years. Because of this predominance of 65 to 70 year olds in the age distribution, every existing age-effect in our study sample is reduced even though the effect is highly reliable and stable. To be able to detect such small effects, one needs statistical power, i.e. an appropriate large sample size. (2) Especially when associated with cognitive, behavioral, or other data collected outside the MRI scanner, connectivity analyses on task-free data are very power-sensitive. In order to be able to present results that also survive the correction for multiple comparisons, we have to use the whole sample of 186 participants. In order to prove our stand that we are dealing with a pure power-problem and not presenting results representing noise inflated by double dipping to a pseudo effect, we repeated all steps from the ICCp analysis to the two versions of connectivity analyses. To ensure the independency of our analyses this time, we blindly divided the sample of 186 participants in two equal halves, confirmed with a *t*-test that the two sub-samples showed no significant age difference, and then used the first half of the sample for the ICCp analysis and the other half of the sample for the connectivity analyses. The results of these additional analyses are presented in an Appendix (Appendix A1 in Supplementary Material) and demonstrate that we did find almost identical results as presented in the main manuscript when using the divided data sets, but a more lenient statistical threshold (*p* < 0.05 uncorrected for multiple comparisons) on the level of the connectivity analyses.

#### Association of the age-related functional loss of connectedness with cognitive performance

The results of the ICCp analysis had highlighted three regions as exhibiting age-related alterations of functional connectedness. To test our hypothesis that these age-effects might as well be related to cognitive functions of our study population, we associated the connection profiles of the three ICCp clusters with the participants' performance scores in the LHAB test-battery.

In order to determine cognitive functions preferably associated with all three regions exhibiting age-related connectedness-effects alike, we used the NeuroSynth database (http://neurosynth.org; Yarkoni et al., 2011).

NeuroSynth is an internet based platform for large-scale, automated synthesis of functional magnetic resonance imaging (fMRI) data extracted from 10,903 published articles, which cover over 386,455 activations (status 23.04.2014). One of the advantages of this platform is its ability to compute posterior probabilities and therefore to quantify the probability that an activated voxel is associated with a particular cognitive function (Yarkoni et al., 2011). We used the NeuroSynth framework to compute the posterior probabilities for the three ICCp clusters' peak voxels of being involved in executive-control (search term: “executive”), working memory (search terms: “wm task” and “working memory”), attention (search term: “attention”), reasoning (search term: “reasoning”), memory (search term: “memory”), and processing speed (search term: “speed”). In combination, the posterior probabilities of all three ICCp peak coordinates loaded highest on working memory (Table 1). Based on this result, we used the participants' performance scores of the Corsi Block Tapping Test as implemented in the Wiener Testsystem (WTS) and the Two-Back Test as implemented in the Testbatterie Aufmerksamkeitspruefung (TAP) to relate them to the connection profile of the ICCp clusters. As both working memory tests showed significant gender effects, we computed the correlations while controlling for gender.

**Table 1:** **Results of the NeuroSynth meta-analysis: Posterior probabilities for different higher cognitive functions**.

**ICC cluster and coordinates**	**Executive control**	**Working memory**	**Attention**	**Reasoning**	**Memory**	**Processing speed**
	“executive”	“wm task” and “working memory”	“attention”	“reasoning”	“memory”	“speed”
Right SFG (+02 +40 +54)	0.56	**0.70**	0.37	**0.78**	0.54	0.52
Right Insula (+34 + 28 +00)	**0.64**	**0.68**	0.57	0.52	0.59	0.58
Left SFG (−20 +32 +52)	0.36	**0.54**	**0.57**	0	**0.54**	0.35

*The table lists the posterior probabilities of the coordinates of the peak voxels of the three ICCp clusters to be involved in executive control, in working memory, attention, memory, and processing speed. The posterior probability quantifies the probability that an activated voxel is associated with a particular cognitive function. In the study presented here, we computed the posterior probabilities for the most important cognitive domains, which are standardly assessed by the LHAB-test battery. The highest posterior probabilities are marked in dark gray; the second highest values are highlighted by a light gray*.

## Results

### Results of the ICC analysis

**Figure 2:**
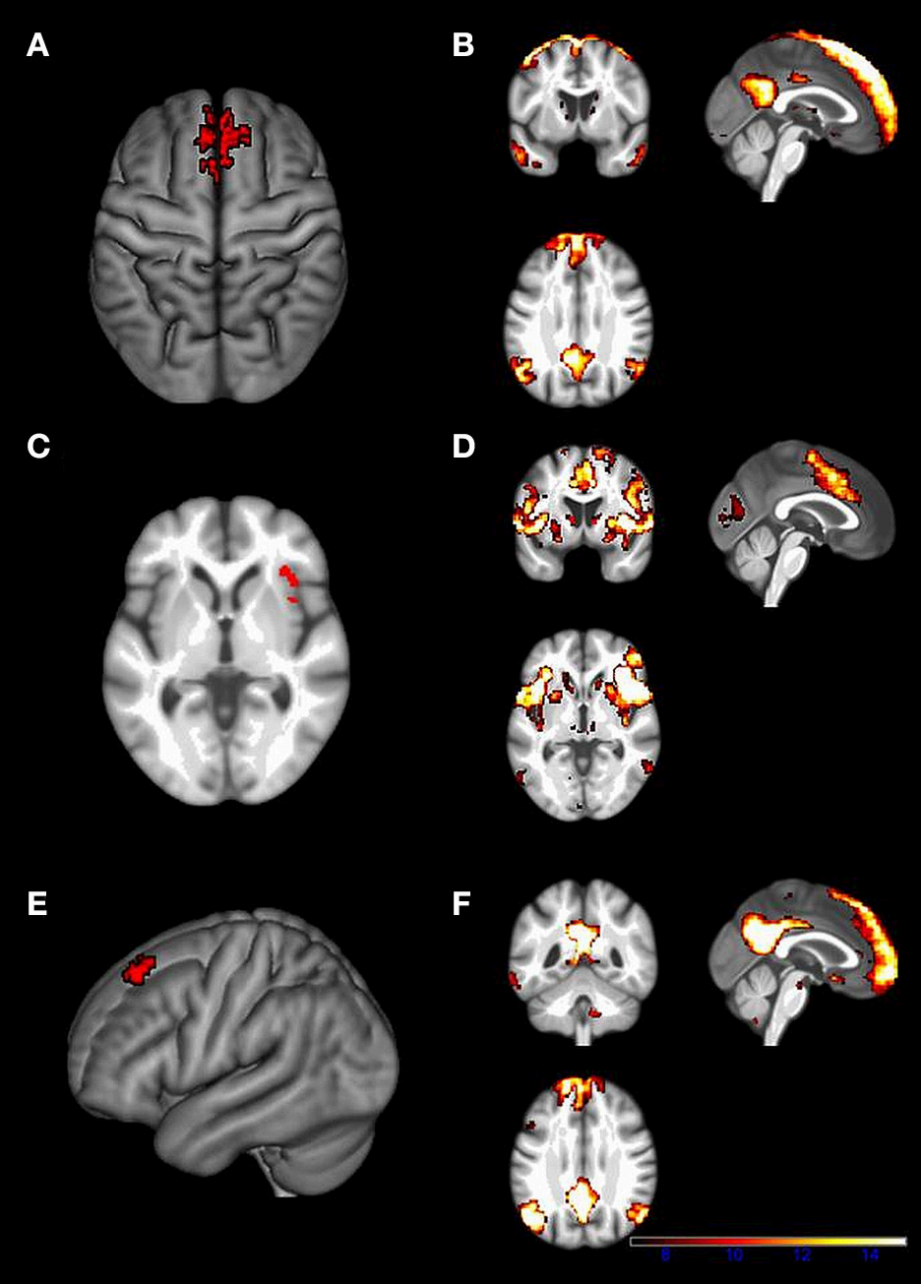
**Locations of the three ICCp clusters and the corresponding SCA cluster maps**. **(A)** ICCp cluster in the right midline part of the superior frontal gyrus. **(B)** The figure displays the corresponding SCA cluster map. The SCA cluster map of the ICCp cluster in the right midline of the superior frontal gyrus identifies it as a part of the DMN. **(C)** ICCp cluster in the right anterior insula. **(D)** The figure displays the corresponding SCA cluster map computed with the ICCp cluster as seed-ROI. The SCA cluster map of the ICCp cluster in the right insula shows the typical pattern of the cingulo-opercular network. **(E)** ICCp cluster in the left superior frontal gyrus. **(F)** The figure displays the corresponding SCA cluster map computed with the ICCp cluster as seed-ROI. The SCA cluster map of the ICCp cluster in the left superior frontal gyrus identifies it as a part of the DMN. All ICCp cluster maps are displayed at a high threshold on voxel-level: *p* < 0.01 uncorrected and a cluster threshold: *p* < 0.05 FWE corrected, the corresponding SCA maps are displayed at a high threshold on voxel-level: *p* < 0.001 FDR corrected and a cluster threshold: *p* < 0.001 FWE corrected.

The ICC analysis revealed three brain regions that exhibited a negative correlation of the ICCp index with the age of the participants. Older subjects showed significantly lower ICCp index values in comparison to the younger subjects: The right superior frontal gyrus (Figure 2A), the right insula (Figure 2C), and the left superior frontal gyrus (Figure 2E), and the (For cluster size and peak voxels, please see Table 2). Interestingly, the ICCp analysis primarily stressed anterior brain regions as exhibiting age-related differences in connectedness.

**Table 2:** **Results of the ICCp analysis**.

**Coordinates of the peak voxel**	**Number of voxels**	**Cluster p-FWE**	**Peak p-FWE**	**Peak p-uncorr**	**Location**
+02 +40 +54	184	0.0000	0.9999	< 0.001	Bilateral medial superior frontal gyrus
+34 +28 +00	68	0.0106	0.0390	< 0.001	Anterior right insula
−20 +32 +52	62	0.0209	1.00	< 0.001	Left superior frontal gyrus

*The table displays the coordinates of the peak voxels, the number of voxels covered by the ICCp cluster, and their location in the brain (high threshold on voxel-level: p < 0.01 uncorrected; cluster threshold: p < 0.05 FWE corrected, only the negative correlation of the ICCp index with age was computed)*.

### Results of the subsequent SCA with the ICCp clusters as seed

To understand better the functional relevance of the ICC clusters and to determine their network affiliation, we computed three SCAs.

- The SCA map for the right superior frontal gyrus ICCp cluster characterized that cluster as belonging to the default mode network (DMN); the SCA map exhibited the classical DMN pattern (Gusnard et al., 2001; Raichle et al., 2001; Greicius et al., 2003) with clusters in the frontal midline reaching into the middle cingulate cortex, in the posterior cingulate cortex as well as in bilateral lateral parietal regions and temporal cortex (Figure 2B).
- The SCA map computed for the ICCp cluster in the right anterior insula (Figure 2D) displayed clusters covering the bilateral thalami and basal ganglia, the bilateral insulae and reaching into the inferior frontal gyri; further clusters were located in the bilateral middle frontal gyri, the bilateral anterior cingulate cortices, the bilateral supramarginal gyrus/inferior parietal lobe regions extending into the superior temporal gyri, and the bilateral calcarine gyri. This specific cluster pattern corresponds to the cingulo-opercular network (CON; Dosenbach et al., 2007; Sadaghiani and D'Esposito, 2014).
- The intrinsic connectivity map for the ICCp cluster in the left superior frontal gyrus likewise showed the characteristic DMN pattern (Figure 2F) with clusters in the bilateral medial part of the superior frontal gyrus, in the bilateral posterior cingulate cortices, the bilateral angular gyri, and the temporal cortices as well as in the hippocampi.

### Results of the low dimensional ICA

We extracted 30 components and 13 of these could be identified as functional meaningful and well established ICNs: C02 cingulo-opercular network (CON); C04 ventral attention network (VAN); C05 auditory network (AUD); C13 anterior default mode network (antDMN); C14 visual network (VIS); C16 left executive control network (LECN); C18 language network (LANG); C20 limbic network (LIMB); C21 sensorimotor network (SMN); C23 higher visual network (highVIS); C25 right executive control network (RECN); C26 dorsal attention network (DAN); C28 posterior default mode network (postDMN); (Figure 1B).

To test for potential within-network age-effects of the 13 ICA components identified as biologically relevant ICNs, we computed the association of each network with age. None of the 13 networks defined by the ICA showed significant age-related degradation of within-network connectivity.

### Results of the connectivity analyses: the connection profiles of the ICCp clusters

**Figure 3:**
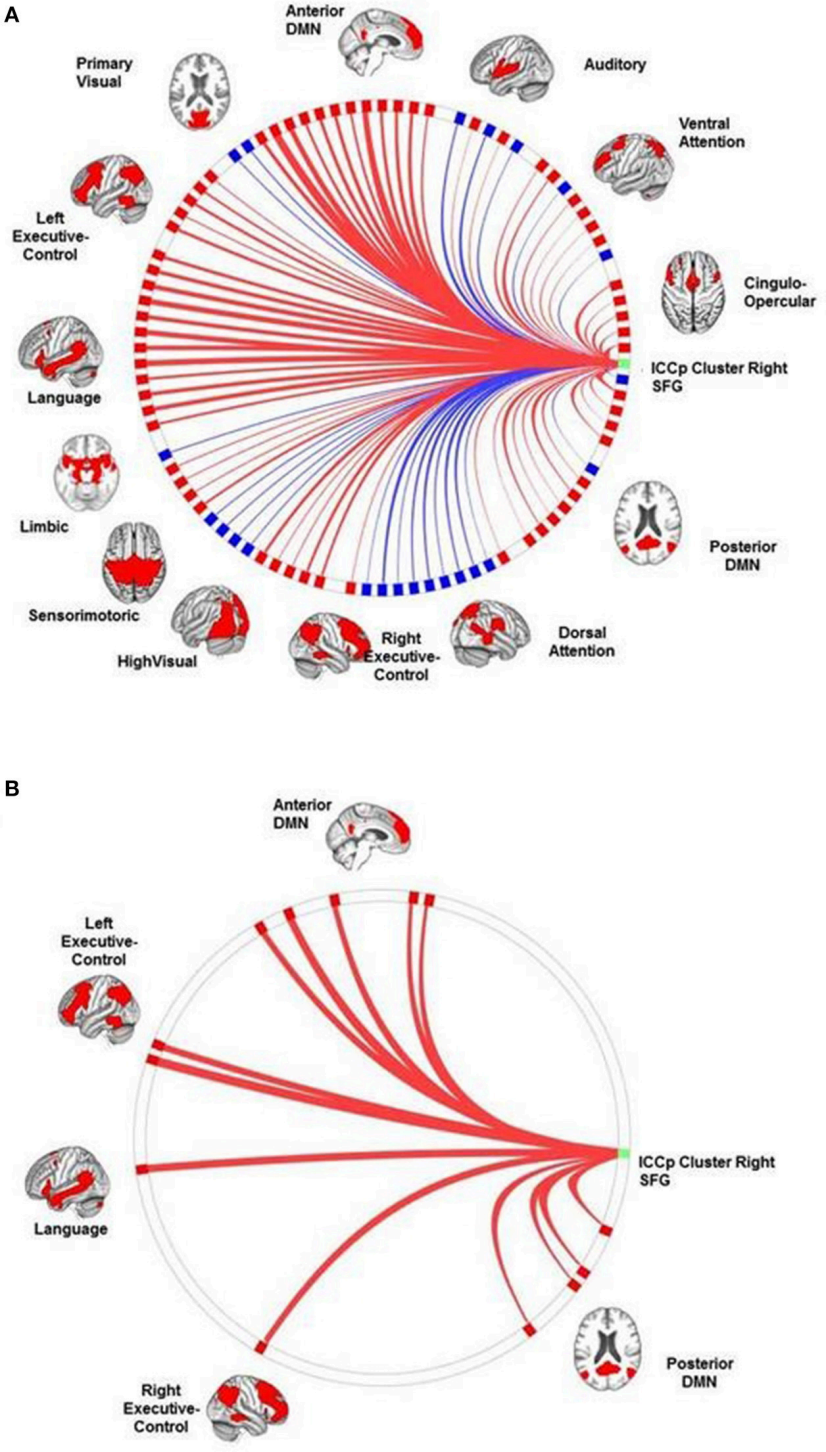
**The connection profiles of the ICCp cluster in the right SFG**. The Panel **(A)** shows the general connection profile of the ICCp cluster located in the right midline of the superior frontal region [*p* < 0.05 FDR corrected (two-sided)]. The blue color indicates negative connections; the red color indicates positive connections. The Panel **(B)** emphasizes the connections and corresponding target ROIs essential for successful performance in the Corsi Block Tapping Test [*p* < 0.05 FDR corrected (two-sided)]. The blue color indicates negative correlations of connection strength with the Corsi Block Tapping Test, the red color indicates positive correlations of connection strength with the Corsi Block Tapping Test. The reader can download a more detailed, high resolution version of these figures as a supplement document from http://journal.frontiersin.org/article/10.3389/fnagi.2016.00086/abstract.

**Figure 4:**
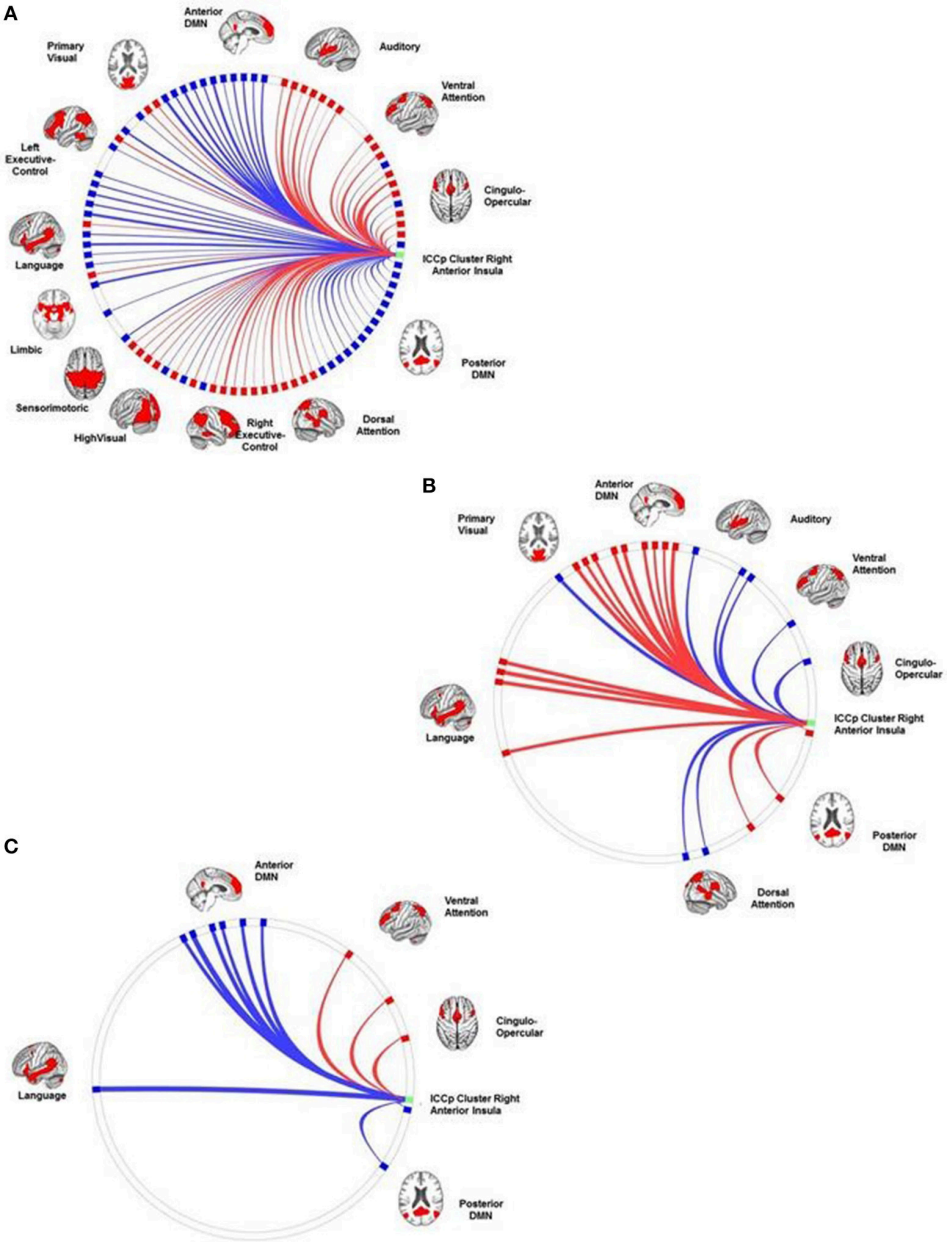
**The connection profiles of the ICCp cluster in the right insula**. The Panel **(A)** shows the general connection profile of the ICCp cluster located in the right anterior insula [*p* < 0.05 FDR corrected (two-sided)]. The blue color indicates negative connections; the red color indicates positive connections. The Panel **(B)** highlights the connections and corresponding target ROIs showing a significant reduction of strength with increasing age [*p* < 0.05 FDR corrected (two-sided)]. The blue color indicates negative correlations of the connection strength with age; the red color indicates positive correlations of the connection strength with age. The Panel **(C)** emphasizes the connections and corresponding target ROIs essential for successful performance in the Corsi Block Tapping Test [*p* < 0.05 FDR corrected (two-sided)]. The red color indicates positive correlations of connection strength with the Corsi Block Tapping Test. The reader can download more detailed and high-resolution figures as a supplement document from http://journal.frontiersin.org/article/10.3389/fnagi.2016.00086/abstract.

**Figure 5:**
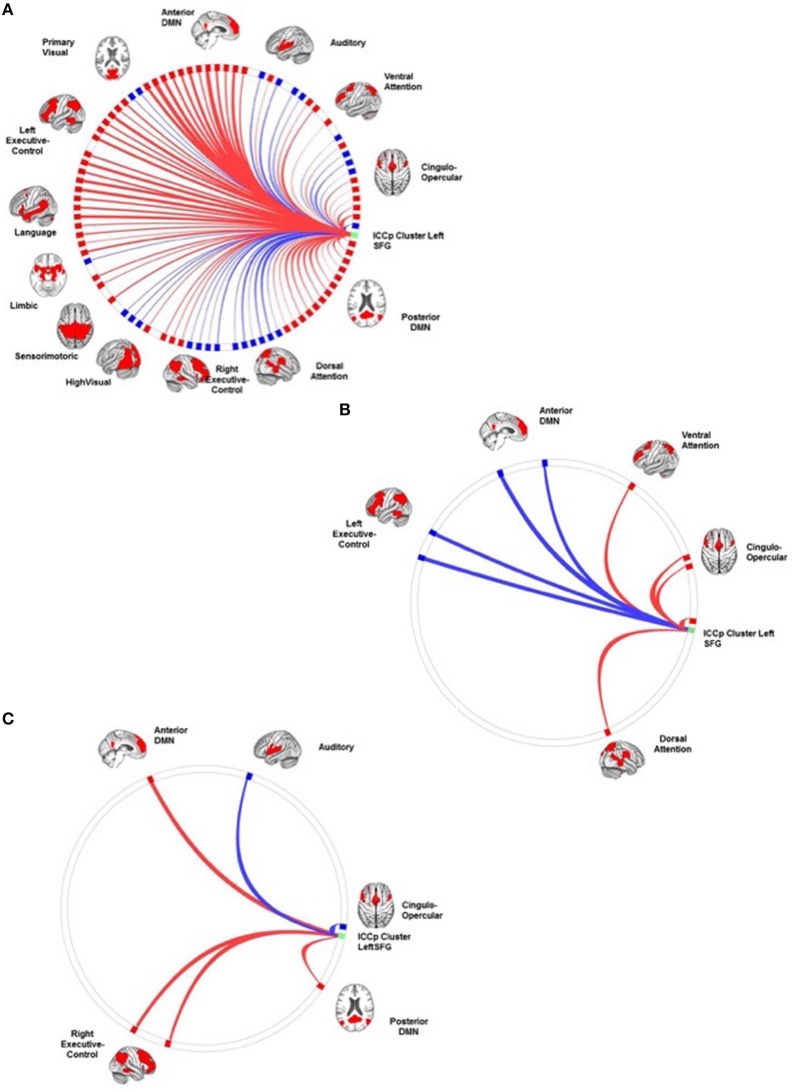
**The connection profiles of the ICCp cluster in the left superior frontal gyrus**. The Panel **(A)** shows the general connection profile of the ICCp cluster located in the left superior frontal gyrus [*p* < 0.05 FDR corrected (two-sided)]. The blue color indicates negative connections; the red color indicates positive connections. The Panel **(B)** highlights the connections and corresponding target ROIs showing a significant reduction of strength with increasing age [*p* < 0.05 FDR corrected (two-sided)]. The blue color indicates negative correlations of the connection strength with age; the red color indicates positive correlations of the connection strength with age. The Panel **(C)** emphasizes the connections and corresponding target ROIs essential for successful performance in the Corsi Block Tapping Test [*p* < 0.05 FDR corrected (two-sided)]. The red color indicates positive correlations of connection strength with the Corsi Block Tapping Test. The reader can download a more detailed, high resolution version of these figures as a supplement document from http://journal.frontiersin.org/article/10.3389/fnagi.2016.00086/abstract.

- ICCp cluster in the right SFG: The SCA had highlighted this cluster as a part of the DMN and its connection profile confirmed this network affiliation since the clusters showed the strongest positive connections with target ROISs of the anterior DMN followed by ROIs of the language network and the LECN. As to be expected for a DMN region, the cluster exhibited significant anti-correlations with the DAN, the SMN, the AUD, and both visual networks (Table 3; Figure 3A).
- ICCp cluster in the right anterior insula: The SCA results had assigned this ICCp cluster as a constituent region of the CON and its connection profile corroborated this network affiliation. The cluster in the right anterior insula exhibited the strongest positive correlations with the ROIs of the CON followed by ROIs of the DAN, VAN, and AUD. As typical for a CON region, the right anterior insula showed significant anti-correlations with the anterior and posterior DMNs and the LANG (Table 4; Figure 4A).
- ICCp cluster in the left SFG: The SCA identified this cluster as functionally belonging to the DMN. Consistent with that affiliation the cluster exhibited the strongest positive connections to the ROIs of the anterior DMN, followed by the ROIs of the LANG, LECN, and postDMN (Table 5; Figure 5A).

**Table 3:** **General connection profile of the right SFG ICCp cluster**.

**Targets**	**Effect size**	**T(185)**	**p-unc**	**p-FDR**		
C13_antDMN_rMedSFG	0.6	36.6	< 0.001	< 0.001		
C13_antDMN_lMedSFG	0.6	35.84	< 0.001	< 0.001		
All_ICCpCluster_LeftSFG	0.52	30.14	< 0.001	< 0.001		
C02_CON_lACC	0.42	25.33	< 0.001	< 0.001		
C13_antDMN_rCrus1	0.43	24.9	< 0.001	< 0.001	Long Distance	Cerebellum
C18_LANG_rCrus2	0.38	22.45	< 0.001	< 0.001	Long Distance	Cerebellum
C18_LANG_lMTG	0.35	21.71	< 0.001	< 0.001	Long Distance	Temporal
C13_antDMN_lANG	0.34	21.49	< 0.001	< 0.001	Long Distance	Parietal
C13_antDMN_lCrus2	0.34	20.46	< 0.001	< 0.001	Long Distance	Cerebellum
C18_LANG_lTriang	0.31	20.38	< 0.001	< 0.001		
C18_LANG_lCrus2	0.3	18.29	< 0.001	< 0.001	Long Distance	Cerebellum
C13_antDMN_lMTG	0.26	18.23	< 0.001	< 0.001	Long Distance	Temporal
C18_LANG_lITG	0.25	17.84	< 0.001	< 0.001	Long Distance	Temporal
C13_antDMN_lPCC	0.27	17.81	< 0.001	< 0.001	Long Distance	Parietal
C13_antDMN_rANG	0.27	16.74	< 0.001	< 0.001	Long Distance	Parietal
C18_LANG_rOrbit	0.25	16.71	< 0.001	< 0.001		
C18_LANG_lPrecun	0.25	16.11	< 0.001	< 0.001	Long Distance	Parietal
C13_antDMN_rPCC	0.24	16.04	< 0.001	< 0.001	Long Distance	Parietal
C02_CON_rACC	0.26	15.95	< 0.001	< 0.001		
C26_DAN_lIPL	−0.21	−15.5	< 0.001	< 0.001	Long Distance	Parietal

*The table lists the 20 target ROIs exhibiting the strongest connections with the source ROI located in the in the right midline of the superior frontal gyrus, the effect sizes, and the t-values. All connections to regions beyond the frontal cortex are defined as long-distance connections and the corresponding target region is specified in the last column of the table*.

**Table 4:** **General connection profile of the right anterior insula ICCp cluster**.

**Targets**	**Effect size**	**T(185)**	**p-unc**	**p-FDR**		
C02_CON_rInsula	0.74	47.95	< 0.001	< 0.001		
C05_AUD_rSTG	0.43	32.41	< 0.001	< 0.001	Long Distance	Temporal
C05_AUD_lSTG	0.38	26.24	< 0.001	< 0.001	Long Distance	Temporal
C04_VAN_bMCC	0.37	26.24	< 0.001	< 0.001		
C02_CON_rACC	0.41	25.62	< 0.001	< 0.001		
C13_antDMN_rPCC	−0.34	−25.2	< 0.001	< 0.001	Long Distance	Parietal
C26_DAN_rSMG	0.39	24.88	< 0.001	< 0.001	Long Distance	Parietal
C13_antDMN_lPCC	−0.36	−24.7	< 0.001	< 0.001	Long Distance	Parietal
C25_RECN_rOper	0.41	24.64	< 0.001	< 0.001		
C26_DAN_lSMG	0.34	22.9	< 0.001	< 0.001	Long Distance	Parietal
C13_antDMN_lANG	−0.34	−22	< 0.001	< 0.001	Long Distance	Parietal
C13_antDMN_rCrus1	−0.3	−21.7	< 0.001	< 0.001	Long Distance	Cerebellum
C04_VAN_rMFG	0.32	21.32	< 0.001	< 0.001		
C02_CON_lInsula	0.3	20.7	< 0.001	< 0.001		
C18_LANG_lPrecun	−0.3	−20.2	< 0.001	< 0.001	Long Distance	Parietal
C13_antDMN_lMTG	−0.27	−19.8	< 0.001	< 0.001	Long Distance	Temporal
All_ICCpCluster_LeftSFG	−0.27	−19.7	< 0.001	< 0.001		
C02_CON_rOper	0.28	18.9	< 0.001	< 0.001		
C18_LANG_rCrus2	−0.23	−18	< 0.001	< 0.001	Long Distance	Cerebellum
C13_antDMN_lMedSFG	−0.25	−17.1	< 0.001	< 0.001		

*The table lists the 20 target ROIs exhibiting the strongest connections with the source ROI located in the in the right anterior insula, the effect sizes, and the t-values. All connections to regions beyond the frontal cortex are defined as long-distance connections and the corresponding target region is specified in the last column of the table*.

**Table 5:** **General connection profile of the left SFG ICCp cluster**.

**Targets**	**Effect size**	**T(185)**	**p-unc**	**p-FDR**		
C13_antDMN_lMedSFG	0.88	41.79	< 0.001	< 0.001		
C13_antDMN_rMedSFG	0.57	35.24	< 0.001	< 0.001		
C13_antDMN_lANG	0.61	32.02	< 0.001	< 0.001	Long Distance	Parietal
All_ICCpCluster_RightSFG	0.52	30.14	< 0.001	< 0.001		
C13_antDMN_rCrus1	0.52	26.85	< 0.001	< 0.001	Long Distance	Cerebellum
C13_antDMN_lPCC	0.54	26.33	< 0.001	< 0.001	Long Distance	Parietal
C13_antDMN_rPCC	0.43	23.49	< 0.001	< 0.001	Long Distance	Parietal
C13_antDMN_lMTG	0.4	22.84	< 0.001	< 0.001	Long Distance	Temporal
C18_LANG_lPrecun	0.42	22.32	< 0.001	< 0.001	Long Distance	Parietal
C16_LECN_lMCC	0.34	21.96	< 0.001	< 0.001		
C18_LANG_lMTG	0.37	21.3	< 0.001	< 0.001	Long Distance	Temporal
C16_LECN_rCrus1b	0.35	21.17	< 0.001	< 0.001	Long Distance	Cerebellum
C28_postDMN_medSFG	0.39	21.02	< 0.001	< 0.001		
C18_LANG_rCrus2	0.36	20.99	< 0.001	< 0.001	Long Distance	Cerebellum
C13_antDMN_rANG	0.36	20.36	< 0.001	< 0.001	Long Distance	Parietal
All_ICCpCluster_RightInsula	−0.27	−19.73	< 0.001	< 0.001		
C26_DAN_rSMG	−0.31	−19.71	< 0.001	< 0.001	Long Distance	Parietal
C16_LECN_lTriang	0.34	19.28	< 0.001	< 0.001		
C04_VAN_lSFG	0.32	19.01	< 0.001	< 0.001		
C13_antDMN_rCereb9	0.25	18.43	< 0.001	< 0.001	Long Distance	Cerebellum

*The table lists the 20 target ROIs exhibiting the strongest connections with the source ROI located in the in the left superior frontal gyrus, the effect sizes, and the t-values. All connections to regions beyond the frontal cortex are defined as long-distance connections and the corresponding target region is specified in the last column of the table*.

### Age-related alterations of the connection-profiles

The ICC analysis had shown that some brain regions exhibited a loss of connectedness with increasing age. In order to get a better understanding of which target regions were most affected, we performed a second connectivity analysis with the aim to elucidate the age-specific association of the connectivity strengths of the ICC source ROIs to the ICA target ROI's.

In contrast to the fine-grained ICC analysis on single voxel level, only two of the ICC clusters showed significant age effects in their connection profiles on ROI-level that also survived the correction for multiple comparisons. In short, the observed age-effects on the connection profiles can be summarized as follows: Age and connection strength showed an inverse relationship.

- With increasing age, the ICCp cluster in the right anterior insula exhibited an overall decrease of connection strength. Both connection types, positively as well as negatively correlated networks or single ROIs were affected by this decrease. Therefore, originally anti-correlated regions like ROI's of the anterior and posterior DMNs now showed a reduction in anti-correlation and therefore a trend to become positively connected with age, while the connection strengths of originally positively correlated ROIs of the CON and the VAN decreased and showed a tendency to become anti-correlated. From the 24 source ROI—target ROI connections showing age-effects, 17 connections were long-distance connections to the temporal (2), parietal (9), and occipital (2) regions of the brain as well as to the cerebellum (3) (Table 6; Figure 4B).
- The same reverse relationship between increasing age and connection strength could be observed for the ICC cluster in the left SFG: Originally, positively connected networks/ROIs now showed a trend to become anti-correlated with increasing age and vice versa, originally anti-correlated networks/ROI's exhibited a trend to become positively correlated. Thus, the originally anti-correlated left SFG became more positively connected with ROIs of the CON, DAN, and VAN in association with age. In contrast, ROIs of the LECN and anterior DMN that originally had showed a clear positive correlation with the ICC cluster now exhibited an decrease in connection strength, i.e., they became more anti-correlated with increasing age. Of the eight source ROI—target ROI connections affected by age four connections were long-distance connections to parietal brain regions (2) and the cerebellum (2) (Table 7; Figure 5B).

**Table 6:** **Connection profile of the right anterior insula ICCp cluster associated with age**.

**Targets**	**Effect size**	**T(184)**	**p-unc**	**p-FDR**		
C13_antDMN_lANG	0.07	4.7	< 0.001	< 0.001	Long Distance	Parietal
All_ICCpCluster_LeftSFG	0.06	4.25	< 0.001	0.002		
C02_CON_rACC	−0.06	−3.98	< 0.001	0.003		
C13_antDMN_lPCC	0.05	3.73	< 0.001	0.006	Long Distance	Parietal
C28_postDMN_medSFG	0.05	3.44	< 0.001	0.014		
C13_antDMN_lMedSFG	0.05	3.31	0.001	0.017		
C04_VAN_rMFG	−0.05	−3.24	0.001	0.017		
C13_antDMN_rPCC	0.04	3.24	0.001	0.017	Long Distance	Parietal
C13_antDMN_rANG	0.05	3.2	0.002	0.017	Long Distance	Parietal
C18_LANG_lMTG	0.04	3.15	0.002	0.018	Long Distance	Temporal
C28_postDMN_lMOC	0.05	3.07	0.002	0.021	Long Distance	Occipital
C18_LANG_lPrecun	0.04	2.93	0.004	0.03	Long Distance	Parietal
C13_antDMN_lCrus2	0.04	2.91	0.004	0.03	Long Distance	Cerebellum
C14_VIS_rCalc	−0.04	−2.84	0.005	0.033	Long Distance	Occipital
C18_LANG_lCrus2	0.04	2.83	0.005	0.033	Long Distance	Cerebellum
C04_VAN_rPrecun	−0.04	−2.8	0.006	0.034	Long Distance	Parietal
C13_antDMN_lMTG	0.04	2.71	0.007	0.04	Long Distance	Temporal
C26_DAN_lSMG	−0.04	−2.68	0.008	0.04	Long Distance	Parietal
C13_antDMN_rCrus1	0.04	2.68	0.008	0.04	Long Distance	cerebellum
C18_LANG_rPrecun	0.04	2.67	0.008	0.04	Long Distance	Parietal
C04_VAN_bMCC	−0.04	−2.64	0.009	0.041		
C13_antDMN_rMedSFG	0.04	2.6	0.01	0.044		
C05_AUD_rThal	−0.03	−2.57	0.011	0.044	Long Distance	Subcortical
C26_DAN_rPrecun	−0.03	−2.57	0.011	0.044	Long Distance	Parietal

*The table lists the connections of the right insula ICCp cluster and the corresponding target ROIs that exhibit an age-related reduction in connection strength or an anti-correlation of age and connection strength [p < 0.05 FDR corrected (two-sided)], the effect sizes, and the t-values. All connections to regions beyond the frontal cortex are defined as long-distance connections and the corresponding target region is specified in the last column of the table*.

**Table 7:** **Connection profile of the left SFG ICCp cluster associated with age**.

**Targets**	**Effect size**	**T(184)**	**p-unc**	**p-FDR**		
All_ICCpCluster_RightInsula	0.06	4.25	0.000033	0.002603		
C02_CON_rInsula	0.06	4.13	0.000054	0.002603		
C13_antDMN_rCrus1	−0.07	−3.72	0.000263	0.007199	Long Distance	Cerebellum
C26_DAN_rSMG	0.06	3.69	0.0003	0.007199	Long Distance	Parietal
C04_VAN_rPrecun	0.04	3.15	0.001881	0.036084	Long Distance	Parietal
C16_LECN_rCrus1b	−0.05	−3.1	0.002255	0.036084	Long Distance	Cerebellum
C13_antDMN_lMedSFG	−0.06	−3.03	0.002802	0.038421		
C16_LECN_lTriang	−0.05	−2.94	0.003689	0.040121		
C02_CON_rACC	0.05	2.93	0.003761	0.040121		

*The table lists the connections of the left SFG ICCp cluster and the corresponding target ROIs that exhibit an age-related reduction in connection strength or an anti-correlation of age and connection strength [p < 0.05 FDR corrected (two-sided)], the effect sizes, and the t-values. All connections to regions beyond the frontal cortex are defined as long-distance connections and the corresponding target region is specified in the last column of the table*.

### Results of the connectivity analysis investigating the association of the three ICCp clusters' connection profiles and working memory performance

We computed the main effect of the participants' performance in two working memory tests (2-back Test and Corsi Block Tapping Test) and the connection profiles of the three ICC clusters while controlling for gender effects. After correction for multiple comparisons, only the Corsi Block Tapping Test exhibited significant associations with the following connection profiles:

- Right superior frontal gyrus: The connection profile of the right SFG associated with the participants' performance in the Corsi Block Tapping Test highlighted only positive long-distance connections with target ROIs primarily belonging to the anterior and posterior DMN and located in the right temporal cortex, the bilateral parietal cortex and the cerebellum. After controlling for age, four positive long-distance connections to the cerebellum (antDMN_rCrus1, LECN_rCrus1b, postDMN_rCereb9) and to the right middle temporal gyrus (postDMN_rMTG) still seemed to be essential for a successful performance (Table 8; Figure 3B).
- Right anterior insula: The number of correct trials in Corsi Block Tapping Test depended on the right anterior insula's preserved negative correlations with ROIs in the frontal cortex, the middle cingulate cortex, the parietal cortex, and the cerebellum. When we repeated the connectivity analysis and additionally controlled for age effects, only a negative correlated connection to a ROI of the antDMN in the right cerebellum crus I showed a significant association with number of correct trials in the Corsi Block Tapping Test (Table 9; Figure 4C).
- Left superior frontal gyrus: Preserved positive long-distance connections to the right middle temporal gyrus and to the bilateral cerebellum (postDMN_rCereb9, antDMN_rCrus1, RECN_lCrus1) as well as preserved negative connections to target ROIs in the right superior temporal gyrus (AUD_rSTG) and to the ICCp cluster located in the right anterior insula were correlated with a successful performance in the Corsi Block Tapping test. After controlling for age, none of these connections survived the correction for multiple comparisons (Table 10; Figure 5C).

**Table 8:** **Connection profile of the right SFG ICCp cluster associated with working memory performance**.

**Targets**	**Effect size**	**T(182)**	**p-unc**	**p-FDR**		
C13_antDMN_rCrus1	0.07	3.87	0.000152	0.00627	Long Distance	Cerebellum
C28_postDMN_rMTG	0.05	3.81	0.000187	0.00627	Long Distance	Temporal
C28_postDMN_rCereb9	0.05	3.8	0.000198	0.00627	Long Distance	Cerebellum
C16_LECN_rCrus1b	0.06	3.72	0.000261	0.00627	Long Distance	Cerebellum
C13_antDMN_lPCC	0.05	3.22	0.001495	0.028691	Long Distance	Parietal
C18_LANG_rITG	0.04	3.13	0.00205	0.028691	Long Distance	Temporal
C25_RECN_lCrus1	0.05	3.12	0.002092	0.028691	Long Distance	Cerebellum
C28_postDMN_lMOC	0.05	3.04	0.002687	0.032242	Long Distance	Occipital
C13_antDMN_rPCC	0.05	2.93	0.003837	0.036335	Long Distance	Parietal
C13_antDMN_lANG	0.05	2.92	0.003925	0.036335	Long Distance	Parietal
C28_postDMN_medSFG	0.05	2.9	0.004163	0.036335		
C13_antDMN_bMCC	0.04	2.78	0.006062	0.046418		
C16_LECN_rCrus1a	0.04	2.76	0.006286	0.046418	Long Distance	Cerebellum

*The table lists the connections and the corresponding target ROIs that have exhibited a significant association (p < 0.05 FDR corrected (two-sided) with the participants' performance in working memory, operationalized by the number of correct sequences in the Corsi Block Tapping Test. All connections to regions beyond the frontal cortex are defined as long-distance connections and the corresponding target region is specified in the last column of the table*.

**Table 9:** **Connection profile of the right anterior insula ICCp cluster associated with working memory performance**.

**Targets**	**Effect size**	**T(182)**	**p-unc**	**p-FDR**		
C13_antDMN_rCrus1^*^	−0.06	−4.49	< 0.0001	0.001	Long Distance	Cerebellum
All_ICCpCluster_LeftSFG^*^	−0.05	−3.85	< 0.0001	0.006		
C18_LANG_rCrus2	−0.05	−3.81	< 0.0001	0.006	Long Distance	Cerebellum
C13_antDMN_rANG^*^	−0.05	−3.65	< 0.0001	0.008	Long Distance	Parietal
C13_antDMN_lMedSFG^*^	−0.05	−3.58	< 0.0001	0.008		
C13_antDMN_lCrus2^*^	−0.05	−3.4	< 0.0001	0.012	Long Distance	Perebellum
C02_CON_rACC^*^	0.05	3.38	< 0.0001	0.012		
C04_VAN_rMFG^*^	0.05	3.2	0.002	0.019		
C13_antDMN_lPCC^*^	−0.05	−3.15	0.002	0.02	Long Distance	Parietal
C13_antDMN_rMedSFG^*^	−0.04	−3.01	0.003	0.027		
C04_VAN_bMCC^*^	0.04	3	0.003	0.027		
C28_postDMN_rCereb9	−0.04	−2.83	0.005	0.041	Long Distance	Cerebellum

*The table lists the connections and the corresponding target ROIs that have exhibited a significant association (p < 0.05 FDR corrected (two-sided) with the participants' performance in working memory, operationalized by the number of correct sequences in the Corsi Block Tapping Test. All connections to regions beyond the frontal cortex are defined as long-distance connections and the corresponding target region is specified in the last column of the table. The asterisks indicate connections that had also shown significant age-effects*.

**Table 10:** **Connection profile of the left SFG ICCp cluster associated with working memory performance**.

**Targets**	**Effect size**	**T(182)**	**p-unc**	**p-FDR**		
All_ICCpCluster_RightInsula^*^	−0.05	−3.85	0.000164	0.015782		
C28_postDMN_rCereb9	0.05	3.26	0.001316	0.039922	Long Distance	Cerebellum
C13_antDMN_rCrus1^*^	0.06	3.17	0.001772	0.039922	Long Distance	Cerebellum
C25_RECN_rMTG	0.05	3.1	0.002208	0.039922	Long Distance	Temporal
C25_RECN_lCrus1	0.04	3.1	0.002227	0.039922	Long Distance	Cerebellum
C05_AUD_rSTG	−0.04	−3.07	0.002495	0.039922	Long Distance	Temporal

*The table lists the connections and the corresponding target ROIs that have exhibited a significant association (p < 0.05 FDR corrected (two-sided) with the participants' performance in working memory, operationalized by the number of correct sequences in the Corsi Block Tapping Test. All connections to regions beyond the frontal cortex are defined as long-distance connections and the corresponding target region is specified in the last column of the table. The asterisks indicate connections that had also shown significant age-effects*.

## Discussion

Increasing age is accompanied by structural and functional brain alterations, which have the potential to affect functions across all cognitive, emotional, and behavioral domains. Previous studies had demonstrated a degradation of the functional architecture of the aging brain as early as in the intrinsically active functional baseline configuration. Based on the assumption that this intrinsically active functional network structure is at least to some degree related to actual goal driven behavior and cognition, our study aimed to investigate which brain regions already exhibit age-related loss of regional inter-connectedness in a task-free condition and how that loss of connectivity might impair behavior and cognition in a large sample of 186 adults in the transition from old to very old age.

In a first step, we performed a purely data-driven ICCp analysis on voxel level with the aim of finding brain regions exhibiting differences of connectedness with increasing age. The ICCp analysis highlighted three regions, all located in the anterior part of the brain.

To determine the ICN affiliation of these regions we then computed a SCA for each of these ICCp clusters. The combination of ICCp analysis and SCA marked two ICNs, the DMN and the cingulo-opercular network, as showing age-related differences, more specifically a decrease of connectedness of their specific constituent regions in the left and right superior frontal gyri, and in the right anterior insula, respectively.

In a third step, we wanted to understand how these ICCp regions were embedded in the functional baseline architecture of the whole brain and which of their target regions were especially affected by these age-related connection changes. For that purpose, we computed three different connectivity analyses, a first analysis to establish the general functional connection profile of each of these ICCp clusters, a second to investigate the influence of higher age on these connection profiles and a third to investigate the possible effects of these age-related alterations on working memory. The results of the first two connectivity analyses can be summed up as follows: Age and connection strength exhibited an anti-correlation: In participants of higher age, the connection profiles of the ICCp clusters showed signs of functional dedifferentiation on the ROI-to-ROI level due to a reduction in connections strength. Especially long-range connections were affected by these reductions in connection strength. The third connectivity analysis demonstrated that these age-effects might have consequences for cognition: The same connections emphasized by the prior connectivity analysis as showing age-related decrease were also stressed as relevant for working memory. Especially preserved long-distance connections from the frontal cortex into the cerebellum, the lateral and medial parts of the parietal cortex to temporal regions seemed to be essential for a successful working memory performance. In the next sections, we will discuss the relevance of our findings in more detail.

### (A) frontal regions seem more affected by age-related alterations of connectedness than regions in the posterior part of the brain

The ICCp analysis on voxel-level mainly highlighted frontal regions as affected in connectedness by increasing age. This finding is remarkable even more because it seems quite paradoxical when combined with the subsequent connectivity analyses that revealed long-distance connections from these frontal regions into the posterior part of the brain as affected by age. This observation inevitably triggers the question: When the source region is affected by a decrease in connectedness should the corresponding target region not also show a decrease in connectedness? To understand the paradox that the ICCp analysis stressed the frontal regions but not their respective target ROIs located in the posterior parts of the brain, one has to look closer at the ICCp index. The value of the ICCp index is determined by the existence of a connection and by the strength of this connection. When only frontal source ROIs show a decrease in the ICCp index but not their posterior target regions then this unilateral phenomenon can only be explained by a decrease in connection strength of the frontal regions while the posterior regions do not show such degradation. Therefore, one might conclude that the results of the ICCp analyses could indicate that the anterior frontal regions of the brain show a different trajectory for connection strength, respectively, a faster decrease in connection strength than the posterior parts of the brain with increasing age.

### (B) the role of the right dorsal anterior insula

The most important finding of our study is the right anterior insula's age-related decrease of connectedness observed for the older participants of the study sample that covered an age range from 65 to 85 years.

The right anterior insula is one of the most influential output hubs of the brain (Sridharan et al., 2008) because it is causally initiating the switching from the brain's internal modus operandi to the external modus operandi vital for all successful goal-directed behavior (Sridharan et al., 2008; Menon and Uddin, 2010; Goulden et al., 2014). The internal modus operandi of the brain is characterized by high activity of the default mode network. Although the default mode network's role in the brain is still not fully understood, the so called “default mode” of the brain can be described as a highly dynamic and exploratory state of the brain characterized by a high variance of synchrony over time (Hellyer et al., 2014). However, for successfully performing goal-directed behavior the brain has to change from the rather unconstrained default mode state into a functionally focused state, which is characterized by increased global synchrony and by a coordinated activity of the control and attention networks of the brain (Hellyer et al., 2014). The right anterior insula initiates this switching from one brain state into the other by signaling the DMN to deactivate and the ECN to activate. A dysfunction of the right anterior insula and as a consequence a disturbed dynamic interplay of ECN and DMN in order to generate and maintain successful goal-directed behavior is associated with schizophrenia, autism, and AHDS (Menon and Uddin, 2010; Moran et al., 2013; Sheffield et al., 2015; Uddin, 2015) but also with cognitive impairments after traumatic brain injury (Bonelle et al., 2012; Jilka et al., 2014).

The SCA demonstrated the ICCp cluster of the right anterior insula as belonging to the cingulo-opercular network, i.e., to one of the prominent control networks constituting the task-positive system of the brain, which has an antagonistic relation to the task-negative system of the brain as presented by the DMN. Bearing this antagonistic intrinsic architecture of the brain in mind, one would expect the anterior insula to show positive correlations with all other networks of the task-positive component while exhibiting negative correlations with the DMN. Nevertheless, we found first indicators of age-related functional degradation in our study sample as early as in the neutral connection profile of the right anterior insula ICCp cluster because it showed preserved negative correlations with the target-ROIs of both default mode networks but only partially the expected positive correlations with the RECN and LECN (Cohen et al., 2014). When we associated the connection profile of the right anterior insula with age there was clear evidence, that the seemingly preserved negative correlations with the anterior and the posterior DMN exhibited a tendency to become weaker, respectively a trend to become more positively correlated with increasing age in our study sample.

Age-related alterations of the functioning of the DMN, especially a failure of the DMN to timely deactivate during cognitive demanding tasks is not a new finding but was already demonstrated by a number of previous studies investigating the aging brain (Grady et al., 2006, 2010; Persson et al., 2007; Meinzer et al., 2012; Gordon et al., 2014). However, the potential causal implication of the right anterior insula in this age-related dysfunctional deactivation of the DMN might present a new insight for the understanding of the disturbed dynamic interaction between DMN and ECN so frequently observed in the aging brain.

### (C) the ICCp clusters in the left and the right superior frontal gyrus: same network affiliation but different functional roles and age-effects

Although the SCA and their general connection profiles identified the ICCp clusters in the right and left SFG as belonging to the same network, the anterior DMN, we found two small but interesting differences between the functional characteristics of these two clusters. The first of these differences might indicate that the DMN does not always act as a functional entity, but that sub-parts of the DMN might have quite different roles depending on the demands of the situation. The right insula and the left SFG ICCp clusters' general connection profiles but also the connection profiles associated with the performance scores in working memory emphasized the importance of strong anti-correlations between these two regions. Likewise, the negative association between the right anterior insula and the left superior frontal gyrus belonged to the connections exhibiting the strongest age-effects, i.e., showing a decrease in anti-correlation. On the other hand, the second DMN cluster in the midline of the superior frontal gyrus, i.e., ICCp cluster in the right SFG, did not exhibit this clear and functionally important anti-correlation with the ICCp cluster in the right anterior insula. When associated with the working memory scores, its connection profile primarily underlined the importance of preserved strong intra-network connections of the DMN for successful task performance, especially to one of the core hubs of the DMN, the posterior cingulate cortex.

Besides their distinct functional roles for working memory, the two SFG ICCp clusters showed spatially different age effects. When associated with age, the connection profile of the left SFG ICCp cluster primarily showed the typical finding of affected long-range connections. In contrast, although the general connection profile for the right SFG ICCp cluster showed a number of long distance connections into the parietal and temporal cortices as well as into the cerebellum, the subsequent association of the connection profile revealed that none of these long-range connections seemed to be significantly affected by age. In the case of the right SFG ICCp cluster the age-related loss of connectedness seems to be a rather local phenomenon.

### D) cognitive relevance of the findings: implications for the aging brain's working memory performance

Previous studies have already associated the successful performance of working memory with the dynamic interaction of three different main ICNs. While the ECN and the cingulo-opercular network activate together and integrate partially during working memory tasks (Cohen et al., 2014), the DMN deactivates (Gordon et al., 2012; Liang et al., 2015).

When associated with the scores for the Corsi Block Tapping Test, the connection profile of the right anterior insula stressed the importance of a clear temporal separation of the DMN and the ECN activity for working memory. Successful performance of working memory was correlated with a high integration of the right anterior insula ICCp cluster into the cingulo-opercular network and the VAN, respectively positive connections with the right dorsolateral prefrontal cortex and the anterior to middle part of the cingulate cortex, and negative correlations with the antDMN and postDMN. The two DMN ICCp clusters showed the reverse relationship: High performance in working memory correlated with high within-network correlation of the DMN, especially with a high positive correlation between the two-midline hubs of the DMN, the ICCp cluster in the right SFG and the target-ROIs in the PCC. This specific network constellation replicated the findings of previous studies, which already demonstrated the relevance of this posterior-anterior midline connection of the DMN for successful behavioral and cognitive performance (Andrews-Hanna et al., 2007).

However, Piccoli et al. (2015) were able to show that the anti-correlated network configuration of the DMN and the two task-positive networks has to be dynamic to a certain degree during working memory tasks. By separating the three different stages during working memory, i.e., encoding of the stimulus—maintaining the stimulus during the delay phase—and retrieval, the authors (Piccoli et al., 2015) demonstrated that the DMN is only fully anti-correlated during the delay phase but shows partial positive correlations with regions of the other two networks during the encoding and retrieval step.

We found that working memory performance in our study group was associated with preserved anti-correlation between the DMN and the cingulo-opercular network. Very cautiously, one might speculate that the aging brain has to preserve this antagonistic network configuration of the three networks characteristic for the delay phase as early as in the intrinsically active functional baseline-configuration in order to be still able to successfully shield the to be maintained stimulus from confounding influences of a not sufficiently deactivated DMN during an actual working memory tasks.

When comparing the working memory performance correlated connection profiles of the three ICCp clusters, there is another interesting feature that should be discussed: The number of long-range connections to the posterior parts of the cerebellum, i.e., the cerebellum crus I and II, which seem relevant for working memory. The relevance of the cerebellum, particularly of the cerebellum lobule VII and the crus I for working memory performance was already established by previous studies (Stoodley et al., 2012; Luis et al., 2015). Luis and colleagues showed that the active involvement of a fronto-parietal-cerebellar network, i.e., the involvement of the right and left ECN with its cerebellar nodes, is important for supporting working memory. Our data seem to complement the findings of Luis et al. (2015) as far as they indicate that also the timely deactivation of cerebellar regions functionally belonging to the DMN is critical for successful working memory performance.

### Limitations

There are some limitations of our study: Within the scope of the LHAB protocol, we only acquire task-free but no task-induced fMRI data, all actual cognitive and behavioral measurements are collected outside the scanner and therefore not directly linked to the brain data from the MRI data acquisition. This fact sets some constraints to the range within which we can elucidate how the aging brain actually performs a certain cognitive task or behavior. However, the aim of the study presented here was not to investigate how the aging brain performs working memory tasks but to investigate which brain regions may exhibit an age-related loss of connectedness in the life-period from old age to very old age and how these age-effects might relate to actual cognition and behavior. This is a valuable research question in itself given the fact that most of the knowledge about the specifics of the aging brain is gained from cross-sectional studies investigating the differences between young adults, usually college students aged from 20 to 30 years, and older adults. Due to these studies, we might better know which age-related differences characterize the older brain in comparison to young brains but understanding age differences on a relatively coarse level does not also imply that we understand the aging processes of the brain that cause these differences. Albeit also cross-sectional, our study aimed to elucidate the age related differences in connectivity on a much more fine-grained level by looking at the intrinsically active baseline configuration in a study sample of healthy older participants aged between 65 to 85 years.

A discussion point still unanswered by the discussion of our findings might be the fact that our data-driven analyses detected age-related alterations in the right anterior insula and in the cingulo-opercular network. We are well aware of the still-ongoing debate about the cingulo-opercular network and its relation to another ICN, the salience network, about the respective functions of these two ICNs and the relation of the anterior insula to them (Seeley et al., 2007; Dosenbach et al., 2007; Sridharan et al., 2008; Power et al., 2011; Sadaghiani and D'Esposito, 2014; Uddin, 2015). Because of analyzing task-free fMRI data and behavioral data only indirectly related to the brain data, we were not able to elucidate the question or to take a stand on the debate. However, the main finding of our study, the age-related functional degradation of the right anterior insula, and its interpretation should not be questioned by this open debate since the influence of the right anterior insula in the brain's switching from the internal to the external modus operandi and the role of the DMN and ECN in this process are insights generally agreed on in the discussion.

### Conclusions

A major contribution of our knowledge of the aging brain either comes from studies comparing young with older adults or from studies investigating pathological aging and using the healthy aging older just as a control group, which is used to highlight the differences between health and disease but is not in the focus of the research interest. The study presented here aimed to understand better which functional changes characterize the healthy aging brain's transition from old age to very old age. We assumed that the state of the intrinsically active functional baseline configuration of the brain might be at least to some degree a limiting factor that determines goal-directed behavior. Therefore, we investigated task-free fMRI data from a large sample of older adults in the transition from old to very old age in order to understand how the intrinsically active functional architecture changes during this transition.

Because of its role as one of the most important output hubs of the brain and its causal influence in the switching from the task-negative into the task-active modus operandi of the brain, the most important finding was the age-related loss of connectedness of the right anterior insula in our study group. Previous studies have already demonstrated that the aging brain shows a reduced capacity to timely deactivate the DMN in comparison to younger adults. Our study might be able contribute to the understanding of this observation by showing that this phenomenon is not merely caused by the DMN exhibiting age-related functional degradation but that the cingulo-opercular network as well might be responsible for the impaired dynamic switching from the task-negative state to the task-positive state.

Additionally, our further results supported the findings of previous studies by showing that the reduction of connection strength and the ICN's merging into each other because of that reduction are not just a characteristic that differentiates young from older brains but an ongoing process still characteristic for the aging brain's transition from old age to very old age.

## Author contributions

AM was responsible for the design of the study, the analysis of the data, and interpretation of data for the work; SM supervised the data acquisition and also the design of the study, analysis of the data, and interpretation of data for the work; LJ supervised the data acquisition and also the design of the study, analysis of the data, and interpretation of data for the work. AM, SM, LJ were all involved in drafting the work or revising it critically for important intellectual content. AM, SM, LJ gave all their final approval of the version to be published. AM, SM, and LJ agree to be accountable for all aspects of the work in ensuring that questions related to the accuracy or integrity of any part of the work are appropriately investigated and resolved.

## Funding

This work was supported by the Velux Stiftung (Project No. 369) and the University Research Priority Program “Dynamics of Healthy Aging”.

### Conflict of interest statement

The authors declare that the research was conducted in the absence of any commercial or financial relationships that could be construed as a potential conflict of interest. The reviewer SB and handling Editor declared their shared affiliation, and the handling Editor states that the process nevertheless met the standards of a fair and objective review
