# Supplementary material for: Small Changes, But Huge Impact? The Right Anterior Insula's Loss of Connection Strength during the Transition of Old to Very Old Age
Source: Front Aging Neurosci. 2016 May 10;8:86. doi: 10.3389/fnagi.2016.00086 (PMC4861722; doi:10.3389/fnagi.2016.00086)
Supplement: Supplementary file 1 [file DataSheet1.docx]

**Appendix A 1: Additional steps to ensure the independency between search and test**

To ensure the independency between search and test, we repeated the ICCp analysis and the subsequent connectivity analyses after dividing the 186 sample used in the main manuscript in two equally numbered and now independent sub-samples. By means of a t-test we confirmed that the two sub-samples did not differ significantly in age (p < 0.001), then repeated the ICC analysis using the data from the first sub-set and the connectivity analysis using the data of the second, independent sub-set. In the following sections we present the results of these additional analyses.

**Table S 1.1: Results of the ICCp analysis for the first half (n = 93) of the study sample**

| **Coordinates of the Peak Voxel** | **Number of Voxels** | **Cluster p-FWE** | **Peak p-FWE** | **Peak p-uncorr** | **Location** |
| --- | --- | --- | --- | --- | --- |
| +02 +40 +54 | 104 | 0.0017 | 1.0000 | 0.0005 | Bilateral medial superior frontal gyrus |
| +38 +22 +02 | 72 | 0.0296 | 0.1051 | 0.0000 | Anterior right insula |

Table S 1.1 shows the results of the ICC analysis, when only the first half of the data is used (peak voxel: p = 0.05 uncorr; cluster extension: p < 0.05 FEW corr.). Again, the right anterior insula and the bilateral midline region of the superior frontal gyrus are highlighted as exhibiting a loss of connectedness with increasing age. Peak voxels and cluster extensions of the version using the divided sample are almost identical with the results of the ICCp analysis when using the entire sample of 186 participants. However, in contrast to the results presented in the main manuscript, the cluster located in left superior frontal is now absent.

**Figure S 1.1: The ICCp clusters from the analysis using the divided sample (n = 93) in comparison to the ICCp clusters using the entire sample of 186 participants.**

**
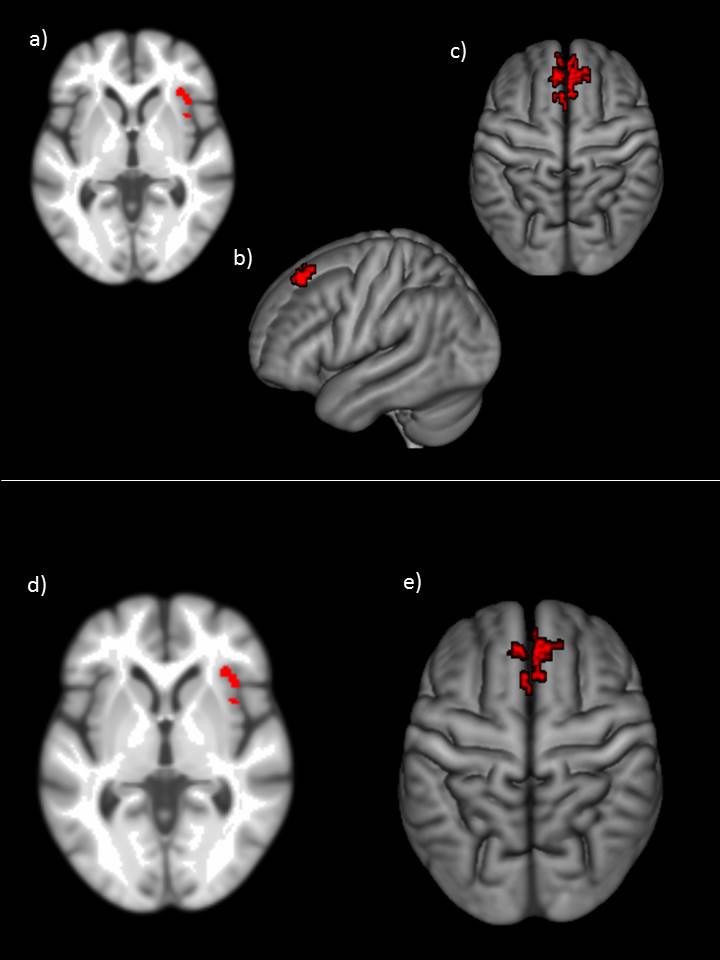
**

In the superior half, the figure S 1.1 shows the ICCp clusters from the analysis using the entire sample; in the inferior part of the illustration we present the ICCp clusters resulting when using the splitted sample of 93 participants. a) ICCp cluster in the right insula (n = 186), b) ICCp cluster located in left superior frontal gyrus (n = 186) c) ICCp cluster in the right midline region of the superior frontal gyrus (n= 186), d) ICCp cluster in the right insula cortex when using the splitted sample of 93 participants and e) ICCp cluster in the right midline part of the superior frontal gyrus when using the splitted sample of 93 participants.

**Table S 1.2: General connection profile of the bilateral SFG ICCp cluster of the divided sample (n = 93) in comparison with the target ROIs of the general connection profile of right SFG ICCp cluster from the analysis using the entire sample (n = 186)**

| **Targets (Sample n = 93)** | **Effect Size** | **T(185)** | **p-unc** | **p-FDR** | **Targets (Sample n = 186) for comparison** |
| --- | --- | --- | --- | --- | --- |
|  |  |  |  |  |  |
| **C13_antDMN_rMedSFG** | 0.5 | 30.87 | < 0.001 | < 0.001 | **C13_antDMN_rMedSFG** |
| **C13_antDMN_lMedSFG** | 0.47 | 27.73 | < 0.001 | < 0.001 | **C13_antDMN_lMedSFG** |
| **C02_CON_lACC** | 0.36 | 21.67 | < 0.001 | < 0.001 | All_ICCpCluster_LeftSFG |
| **C13_antDMN_rCrus1** | 0.36 | 21.17 | < 0.001 | < 0.001 | **C02_CON_lACC** |
| **C18_LANG_rCrus2** | 0.31 | 19.08 | < 0.001 | < 0.001 | **C13_antDMN_rCrus1** |
| **C13_antDMN_lCrus2** | 0.3 | 18.06 | < 0.001 | < 0.001 | **C18_LANG_rCrus2** |
| **C18_LANG_lMTG** | 0.27 | 17.22 | < 0.001 | < 0.001 | **C18_LANG_lMTG** |
| **C13_antDMN_lANG** | 0.26 | 16.64 | < 0.001 | < 0.001 | **C13_antDMN_lANG** |
| **C18_LANG_lTriang** | 0.25 | 16.55 | < 0.001 | < 0.001 | **C13_antDMN_lCrus2** |
| **C18_LANG_rOrbit** | 0.24 | 16.41 | < 0.001 | < 0.001 | **C18_LANG_lTriang** |
| **C18_LANG_lCrus2** | 0.27 | 16.23 | < 0.001 | < 0.001 | **C18_LANG_lCrus2** |
| **C02_CON_rACC** | 0.26 | 15.58 | < 0.001 | < 0.001 | **C13_antDMN_lMTG** |
| **C18_LANG_lITG** | 0.21 | 15.32 | < 0.001 | < 0.001 | **C18_LANG_lITG** |
| **C13_antDMN_lMTG** | 0.22 | 14.87 | < 0.001 | < 0.001 | **C13_antDMN_lPCC** |
| **C13_antDMN_rANG** | 0.23 | 14.36 | < 0.001 | < 0.001 | **C13_antDMN_rANG** |
| **C26_DAN_lIPL** | -0.19 | -14.29 | < 0.001 | < 0.001 | **C18_LANG_rOrbit** |
| **C13_antDMN_lPCC** | 0.21 | 13.95 | < 0.001 | < 0.001 | C18_LANG_lPrecun |
| C18_LANG_rMTG | 0.2 | 13.88 | < 0.001 | < 0.001 | **C13_antDMN_rPCC** |
| **C13_antDMN_rPCC** | 0.2 | 13.34 | < 0.001 | < 0.001 | **C02_CON_rACC** |
| C25_RECN_lCrus1 | 0.21 | 13.14 | < 0.001 | < 0.001 | **C26_DAN_lIPL** |

On the left part, the table S 1.2 shows the results of the connectivity analysis when using the ICCp cluster located in the right SFG as the source ROI and the 93 clusters from the low dimensional ICA as target ROIs (p < 0.05 FDR (seed-level), two-sided). For comparison, the results of the connectivity analysis using the entire sample of 186 participants are listed on the right side of the table, identical target-ROIs of both versions are highlighted by bold letters.

**Table S 1.3: General connection profile of the right anterior insula ICCp cluster of the divided sample (n = 93) in comparison with the target ROIs of the general connection profile of right insula ICCp cluster from the analysis using the entire sample (n = 186)**

| **Targets (Sample n = 93)** | **Effect Size** | **T(185)** | **p-unc** | **p-FDR** | **Targets (Sample n = 186 ) for comparison** |
| --- | --- | --- | --- | --- | --- |
|  |  |  |  |  |  |
| **C02_CON_rInsula** | 0.74 | 48.02 | < 0.001 | < 0.001 | **C02_CON_rInsula** |
| **C05_AUD_rSTG** | 0.44 | 33.93 | < 0.001 | < 0.001 | **C05_AUD_rSTG** |
| **C05_AUD_lSTG** | 0.39 | 27.44 | < 0.001 | < 0.001 | **C05_AUD_lSTG** |
| **C04_VAN_bMCC** | 0.38 | 27.06 | < 0.001 | < 0.001 | **C04_VAN_bMCC** |
| **C02_CON_rACC** | 0.42 | 26.08 | < 0.001 | < 0.001 | **C02_CON_rACC** |
| **C26_DAN_rSMG** | 0.4 | 25.96 | < 0.001 | < 0.001 | **C13_antDMN_rPCC** |
| **C13_antDMN_rPCC** | -0.34 | -25.46 | < 0.001 | < 0.001 | **C26_DAN_rSMG** |
| **C13_antDMN_lPCC** | -0.36 | -25.19 | < 0.001 | < 0.001 | **C13_antDMN_lPCC** |
| **C25_RECN_rOper** | 0.4 | 24.13 | < 0.001 | < 0.001 | **C25_RECN_rOper** |
| **C26_DAN_lSMG** | 0.34 | 23.54 | < 0.001 | < 0.001 | **C26_DAN_lSMG** |
| **C13_antDMN_lANG** | -0.34 | -22.33 | < 0.001 | < 0.001 | **C13_antDMN_lANG** |
| **C04_VAN_rMFG** | 0.33 | 21.59 | < 0.001 | < 0.001 | **C13_antDMN_rCrus1** |
| **C13_antDMN_rCrus1** | -0.3 | -21.56 | < 0.001 | < 0.001 | **C04_VAN_rMFG** |
| **C18_LANG_lPrecun** | -0.31 | -20.63 | < 0.001 | < 0.001 | **C02_CON_lInsula** |
| **C02_CON_lInsula** | 0.3 | 20.51 | < 0.001 | < 0.001 | **C18_LANG_lPrecun** |
| **C13_antDMN_lMTG** | -0.27 | -19.95 | < 0.001 | < 0.001 | **C13_antDMN_lMTG** |
| **C02_CON_rOper** | 0.28 | 18.46 | < 0.001 | < 0.001 | All_ICCpCluster_LeftSFG |
| **C18_LANG_rCrus2** | -0.23 | -17.56 | < 0.001 | < 0.001 | **C02_CON_rOper** |
| C18_LANG_rPrecun | -0.24 | -17.39 | < 0.001 | < 0.001 | **C18_LANG_rCrus2** |
| C04_VAN_lMFG | 0.24 | 17.29 | < 0.001 | < 0.001 | C13_antDMN_lMedSFG |

On the left, the table S 1.3 shows the results of the connectivity analysis when using the ICCp cluster located in the right insula as the source ROI and the 93 clusters from the low dimensional ICA as target ROIs (p < 0.05 FDR (seed-level), two-sided). For comparison, the results of the connectivity analysis using the entire sample of 186 participants are listed on the right side of the table, identical target ROIs of both versions are highlighted by bold letters.

**Table S 1.4: Associated with age: Connection profile of the bilateral SFG ICCp cluster of the divided sample (n = 93) in comparison with the target ROIs of the general connection profile of right SFG ICCp cluster from the analysis using the entire sample (n = 186)**

| **Targets (Sample n = 93)** | **Effect Size** | **T(184)** | **p-unc** | **p-FDR** | **Targets (Sample n = 186) for comparison (p < 0.05 uncorr)** |
| --- | --- | --- | --- | --- | --- |
|  |  |  |  |  |  |
| **C18_LANG_lTriang** | -0.03 | -1.8 | 0.07287 | 0.833529 | **C18_LANG_lTriang** |
| **C16_LECN_lMCC** | -0.02 | -1.75 | 0.08177 | 0.833529 | **C13_antDMN_lANG** |
| C28_postDMN_lMOC | -0.03 | -1.71 | 0.088253 | 0.833529 | **C13_antDMN_rANG** |
| **C13_antDMN_lANG** | -0.03 | -1.67 | 0.096522 | 0.833529 | C13_antDMN_rMedSFG |
| C13_antDMN_lOrbit | -0.02 | -1.67 | 0.09707 | 0.833529 | **C13_antDMN_rCrus1** |
| **C16_LECN_rCrus1b** | -0.03 | -1.63 | 0.105417 | 0.833529 | **C18_LANG_lITG** |
| **C13_antDMN_rANG** | -0.03 | -1.51 | 0.13366 | 0.833529 | **C18_LANG_rCrus2** |
| C16_LECN_rCrus1a | -0.02 | -1.5 | 0.134298 | 0.833529 | **C16_LECN_rCrus1b** |
| **C13_antDMN_bMCC** | -0.02 | -1.5 | 0.136025 | 0.833529 | C13_antDMN_lMedSFG |
| C16_LECN_lOrbit | -0.02 | -1.48 | 0.141816 | 0.833529 | **C25_RECN_lCrus1** |
| C16_LECN_lTriang | -0.02 | -1.44 | 0.150556 | 0.833529 | **C18_LANG_rOrbit** |
| **HalfICCpCluster_RightInsula** | 0.02 | 1.44 | 0.151294 | 0.833529 | **C13_antDMN_bMCC** |
| **C18_LANG_lITG** | -0.02 | -1.4 | 0.163477 | 0.833529 | All_ICCpCluster_LeftSFG |
| C02_CON_rACC | 0.03 | 1.39 | 0.165201 | 0.833529 | **C13_antDMN_lPCC** |
| **C25_RECN_lCrus1** | -0.03 | -1.34 | 0.182267 | 0.833529 | **All_ICCPCluster_RightInsula** |
| **C18_LANG_rOrbit** | -0.02 | -1.29 | 0.197493 | 0.833529 | **C28_postDMN_medSFG** |
| C02_CON_lInsula | -0.02 | -1.28 | 0.20277 | 0.833529 | **C16_LECN_lMCC** |
| **C18_LANG_rCrus2** | -0.03 | -1.26 | 0.209866 | 0.833529 |  |
| **C13_antDMN_rCrus1** | -0.03 | -1.25 | 0.212779 | 0.833529 |  |
| C28_postDMN_rCereb9 | -0.02 | -1.25 | 0.213671 | 0.833529 |  |
| **C28_postDMN_medSFG** | -0.02 | -1.23 | 0.218565 | 0.833529 |  |
| C26_DAN_rSMG | 0.02 | 1.23 | 0.218754 | 0.833529 |  |
| C28_postDMN_rMTG | -0.02 | -1.23 | 0.22061 | 0.833529 |  |
| C28_postDMN_lMTG | -0.02 | -1.23 | 0.22128 | 0.833529 |  |
| **C13_antDMN_lPCC** | -0.02 | -1.22 | 0.224552 | 0.833529 |  |

On the left, the table S 1.4 shows the connection profile of the ICCp cluster located in the right midline part of the superior frontal gyrus when associated with age. As well as when we computed the age associated connection profile of its 186 sample equivalent in the superior frontal gyrus, there were no connections which did survive the FDR correction, indicating that the age-related loss of connectedness highlighted by the ICCp analysis might rather be a local phenomenon and not affecting the existing long-distance connections. For comparison, the results of the connectivity analysis using the entire sample of 186 participants are listed on the right side of the table, identical target ROIs of both versions are highlighted by bold letters.

**Table S 1.5: Associated with age: Connection profile of the right anterior insula ICCp cluster of the divided sample (n = 93) in comparison with the target ROIs of the general connection profile of right anterior insula ICCp cluster from the analysis using the entire sample (n = 186)**

| **Targets (Sample n = 93)**  **p < 0.05 uncorr** | **Effect Size** | **T(184)** | **p-unc** | **p-FDR** | **Targets (Sample n = 186) for comparison (p < 0.05 FDR)** |
| --- | --- | --- | --- | --- | --- |
|  |  |  |  |  |  |
| **C28_postDMN_medSFG** | 0.04 | 2.89 | **0.0043** | 0.38148 | **C13_antDMN_lANG** |
| **C13_antDMN_lANG** | 0.06 | 2.57 | **0.01102** | 0.38148 | **All_ICCpCluster_LeftSFG** |
| **C04_VAN_rPrecun** | -0.03 | -2.4 | **0.01739** | 0.38148 | **C02_CON_rACC** |
| **C18_LANG_lMTG** | 0.04 | 2.29 | **0.02333** | 0.38148 | **C13_antDMN_lPCC** |
| **C14_VIS_rCalc** | -0.03 | -2.26 | **0.02524** | 0.38148 | **C28_postDMN_medSFG** |
| **C13_antDMN_lMedSFG** | 0.04 | 2.17 | **0.03116** | 0.38148 | **C13_antDMN_lMedSFG** |
| **C13_antDMN_rANG** | 0.04 | 2.11 | **0.03658** | 0.38148 | **C04_VAN_rMFG** |
| **C28_postDMN_lMOC** | 0.04 | 2.05 | **0.04158** | 0.38148 | **C13_antDMN_rPCC** |
| C25_RECN_rMCC | 0.03 | 1.99 | **0.04822** | 0.38148 | **C13_antDMN_rANG** |
| **C13_antDMN_rMedSFG** | 0.03 | 1.97 | 0.050784 | 0.381475 | ***C18_LANG_lMTG*** |
| **C18_LANG_lCrus2** | 0.03 | 1.91 | 0.057058 | 0.381475 | ***C28_postDMN_lMOC*** |
| **C02_CON_rACC** | -0.05 | -1.89 | 0.060732 | 0.381475 | ***C18_LANG_lPrecun*** |
| C13_antDMN_bMCC | 0.02 | 1.88 | 0.061388 | 0.381475 | ***C13_antDMN_lCrus2*** |
| **C13_antDMN_lPCC** | 0.04 | 1.84 | 0.067014 | 0.381475 | ***C14_VIS_rCalc*** |
| **C13_antDMN_lCrus2** | 0.03 | 1.83 | 0.068307 | 0.381475 | ***C18_LANG_lCrus2*** |
| C14_VIS_lCalc | -0.03 | -1.82 | 0.070143 | 0.381475 | ***C04_VAN_rPrecun*** |
| C25_RECN_rMTG | 0.03 | 1.76 | 0.079286 | 0.381475 | *C13_antDMN_lMTG* |
| C18_LANG_lTriang | 0.03 | 1.73 | 0.084534 | 0.381475 | *C26_DAN_lSMG* |
| **C18_LANG_rPrecun** | 0.03 | 1.73 | 0.084776 | 0.381475 | *C13_antDMN_rCrus1* |
| C18_LANG_rMTG | 0.03 | 1.73 | 0.085136 | 0.381475 | ***C18_LANG_rPrecun*** |
| C05_AUD_rThal | -0.02 | -1.73 | 0.085968 | 0.381475 | *C04_VAN_bMCC* |
| **C18_LANG_lPrecun** | 0.04 | 1.71 | 0.088342 | 0.381475 | ***C13_antDMN_rMedSFG*** |
| C20_LIMB_lCUN | -0.02 | -1.64 | 0.102108 | 0.400859 | *C05_AUD_rThal* |
| **C26_DAN_rPrecun** | -0.03 | -1.64 | 0.103037 | 0.400859 | ***C26_DAN_rPrecun*** |

The table S 1.5 shows the connection profile of the ICCp cluster located in the right anterior insula when associated with age (the statistical threshold p = 0.05 uncorr is marked by bold letters). For comparison, the results of the connectivity analysis using the entire sample of 186 participants are listed on the right side of the table, identical target-ROIs of both versions are highlighted by bold letters.

**Figure S 1.2: The general connection profile and the connection profile associated with age of the right insula ICCp cluster when using the divided sample versus the general connection profile and the connection profile associated with age of the right insula ICCp cluster when using the entire sample.**

**
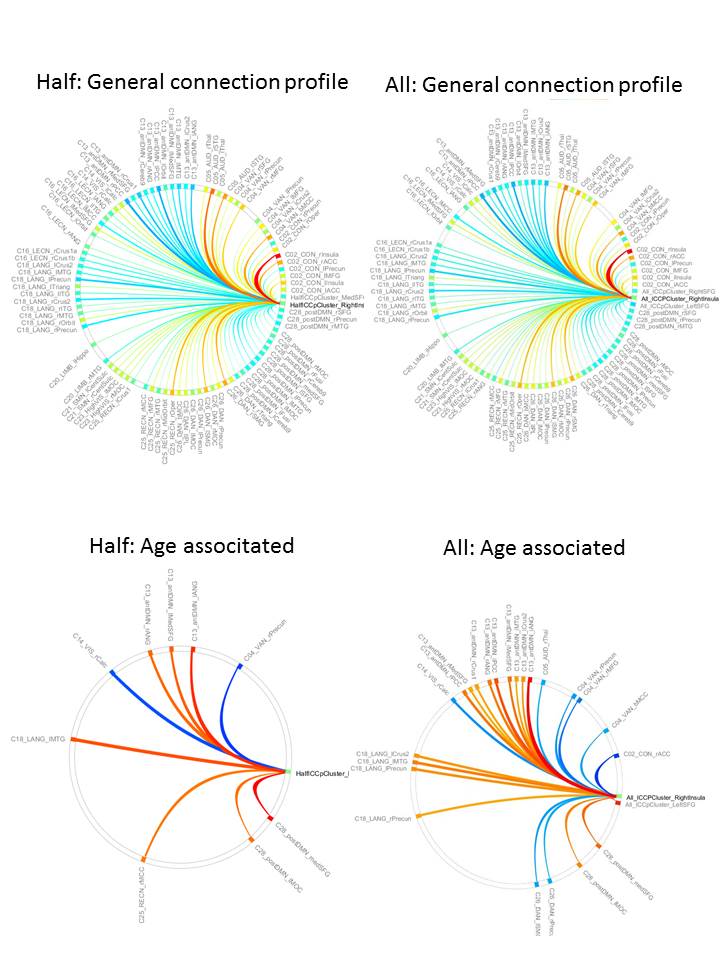
**
