# Supplementary figures and images for: Small Changes, But Huge Impact? The Right Anterior Insula's Loss of Connection Strength during the Transition of Old to Very Old Age
Source: Front Aging Neurosci. 2016 May 10;8:86. doi: 10.3389/fnagi.2016.00086 (PMC4861722; doi:10.3389/fnagi.2016.00086)

4c

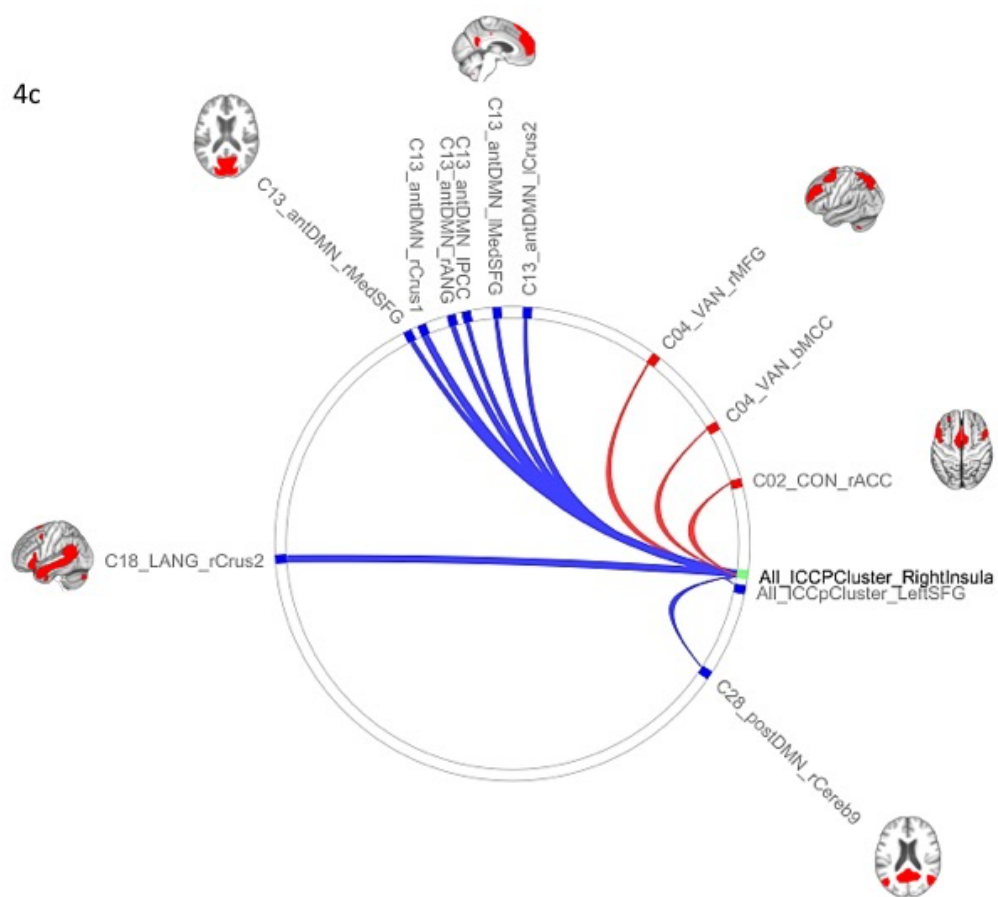



5c

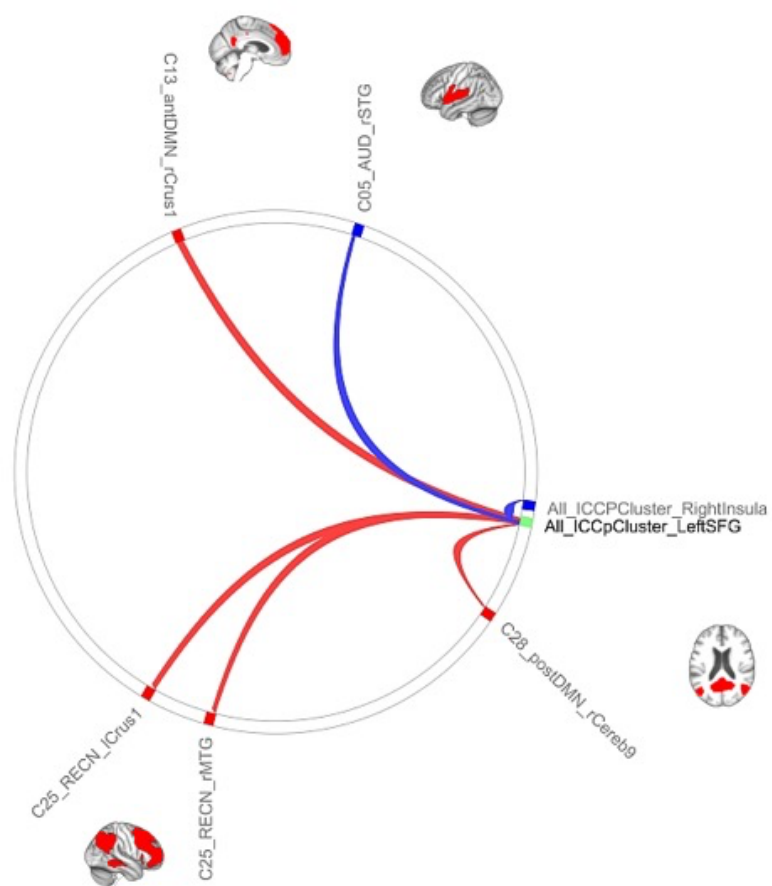

Supplement: Supplementary file 2 [file Images3-5.pdf]
